# Predictive Modeling of High-Entropy Alloys and Amorphous Metallic Alloys Using Machine Learning

**DOI:** 10.1021/acs.jcim.4c00873

**Published:** 2024-10-01

**Authors:** Son Gyo Jung, Guwon Jung, Jacqueline M. Cole

**Affiliations:** † Cavendish Laboratory, Department of Physics, University of Cambridge, J. J. Thomson Avenue, Cambridge CB3 0HE, U.K.; ‡ ISIS Neutron and Muon Source, STFC Rutherford Appleton Laboratory, Harwell Science and Innovation Campus, Didcot, Oxfordshire OX11 0QX, U.K.; § Research Complex at Harwell, Rutherford Appleton Laboratory, Harwell Science and Innovation Campus, Didcot, Oxfordshire OX11 0FA, U.K.; ∥ Scientific Computing Department, STFC Rutherford Appleton Laboratory, Harwell Science and Innovation Campus, Didcot, Oxfordshire OX11 0QX, U.K.

## Abstract

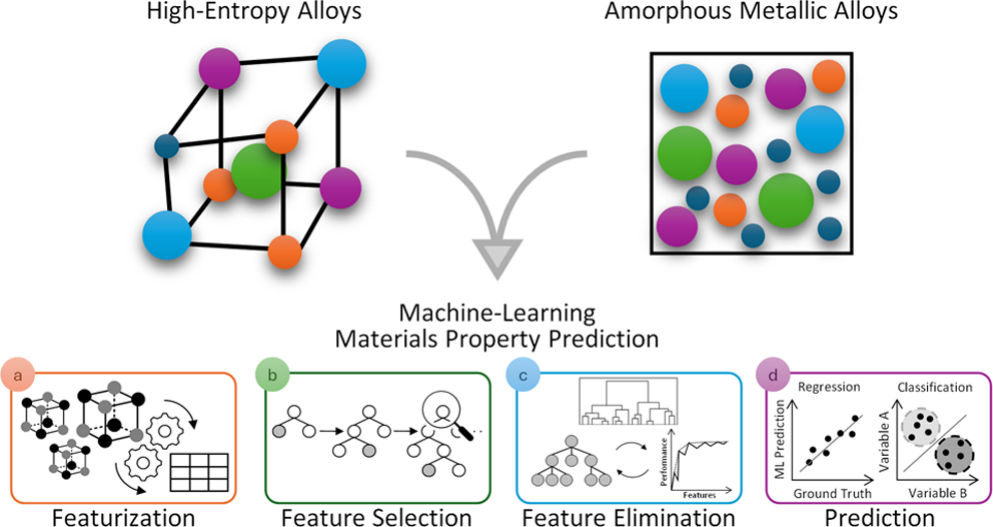

High entropy alloys and amorphous metallic alloys represent two distinct classes of advanced alloy materials, each with unique structural characteristics. Their emergence has garnered considerable interest across the materials science and engineering communities, driven by their promising properties, including exceptional strength. However, their extensive compositional diversity poses substantial challenges for systematic exploration, as traditional experimental approaches and high-throughput calculations struggle to efficiently navigate this vast space. While the recent development in data-driven materials discovery could potentially help, such efforts are hindered by the scarcity of comprehensive data and the lack of robust predictive tools that can effectively link alloy composition with specific properties. To address these challenges, we have deployed a machine-learning-based workflow for feature selection and statistical analysis to afford predictive models that accelerate the data-driven discovery and optimization of these advanced materials. Our methodology is validated through two case studies: (i) a regression analysis of the bulk modulus, and (ii) a classification analysis based on glass-forming ability. The Bayesian-optimized regression model trained for the prediction of bulk modulus achieved an *R*^2^ of 0.969, an mean absolute error (MAE) of 3.958 GPa, and an root mean square error (RMSE) of 5.411 GPa, while our classification model for predicting glass-forming ability achieved an F1-score of 0.91, an area-under-the-curve of the receiver-operating-characteristic curve of 0.98, and an accuracy of 0.91. Furthermore, by leveraging a wide array of chemical data from diverse literature sources, we have successfully predicted a broad range of properties. This success underscores the efficacy of our modeling approach and emphasizes the importance of a comprehensive feature analysis and judicious feature selection strategy over a mere reliance on complex modeling techniques.

## 1 Introduction

A high-entropy alloy (HEA) is a novel class of material that contains five or more principal elements, each contributing between 5 and 35% to the overall alloy composition.^1^ HEAs are often referred to as multiprincipal element alloys, multicomponent alloys, and compositionally complex alloys. This diversification in chemical composition can lead to high configurational entropy, which is postulated to favor the stabilization of solid-solution (SS) phases. HEAs challenge the conventional alloy design that typically relies on one or two principal elements supplemented by minor dopants of other elements. The emergence of HEAs has rapidly expanded the frontiers of materials science, driven by their potential for a wide array of applications. This is attributed to their broad range of mechanical properties, including high strength,^2^ ductility,^3^ corrosion resistance,^4,5^ among others.^6,7^ The desire to enhance such properties underscores their significance and explains the growing interest in exploring these materials for advanced engineering applications.

Amorphous metallic alloys (AMAs) represent another class of alloys that are of material interest in this study. They are commonly referred to as metallic glasses, glassy alloys, or noncrystalline alloys. AMAs are defined by their lack of long-range atomic order, in contrast to their crystalline counterparts. Their amorphous structure is typically obtained via rapid solidification of alloy constituents from gaseous or liquid states. Such a rapid cooling or quenching mechanism is critical because it precludes the atoms within the molten alloy from adopting a crystalline arrangement, whereby the atoms are “frozen” in a liquid-like, metastable configuration during the fast rate of solidification.^8^ This distinctive structural attribute underpins the unique mechanical properties of AMAs, including high strength,^9−11^ excellent wear and corrosion resistance,^12^ among others.^13,14^ Such properties arise because noncrystalline structures lack the grain boundaries and dislocations that are present in their crystalline material counterparts. The absence of such defects in these materials contributes to their high mechanical strength and resistance to deformation. The ongoing research into the production, characterization, and application of AMAs continues to expand their use and potential in advanced engineering and technologies.

The development of HEAs and AMAs has traditionally been guided by experimental research via a laborious “trial-and-error” methodology. This conventional approach demands extensive time commitments and incurs high operational costs, all the while requiring deep domain expertize to navigate the expansive compositional landscape. It is becoming increasingly clear that the design of novel alloys could greatly benefit from adopting strategies that enable the systematically targeted creation of new materials based on desired properties, with machine learning (ML) poised to play a pivotal role in this paradigm shift.

The integration of ML into the materials-design process has led to data-driven materials-prediction methods that can be streamlined through computational simulations of their properties.^15^ This has been demonstrated by a series of studies in various domains, which include the materials prediction of high-entropy ceramics,^16^ organic molecules for light-emitting diodes,^17^ light-harvesting molecules for photovoltaic devices,^18,19^ magnetic refrigerants for storing hydrogen,^20,21^ and band gap materials.^22^ These examples demonstrate the efficacy of data-driven design-to-device pipelines that expedite the exploration of diverse chemical and feature spaces at a pace unattainable through conventional experimental “trial-and-error” synthetic processes. In doing so, it opens the door to considerable operational efficiencies, focusing resource-intensive experimental efforts on the most viable material candidates. This shift not only accelerates the pace of engineering innovation but also fosters a more economically sustainable model for material discovery, development and materials characterization.^23−26^

Within the research domain of HEAs, there has been a discernible shift toward integrating computational methodologies into pipelines for data-driven materials discovery, whose data are predominantly sourced by high-throughput calculations. For example, Lederer et al.^27^ implemented a high-throughput computational methodology to approximate the transition temperature of SS phases of HEAs, integrating *ab initio* energies into a mean field statistical mechanical framework. The underlying approach was corroborated by Monte Carlo simulations, ensuring robustness and accuracy in their estimations. Another notable contribution to this field was made by Senkov et al.,^28^ who employed a combinatorial strategy aimed at accelerating the exploration of HEAs that exhibit SS phases. This approach employed the calculated phase diagram method to evaluate structural candidates for metal alloys. A key finding in their study challenges a foundational hypothesis of HEAs; specifically, it was determined that the stability of SS phases in HEAs does not necessarily increase as a function of the number of alloying elements. This observation stands in contrast to the traditional belief that heightened configurational entropy, resultant from an increased number of alloy elements, would inherently augment the stability of disordered SS phases. More recently, there has been efforts to study HEAs using ML techniques.^29−31^ However, the predominance of such studies, some of which depend on experimental data sets,^32,33^ faces challenges due to the limited availability of HEA data. This data scarcity has necessitated a more focused research approach in the HEA field, often concentrating on specific alloy systems, as reflected in various studies.^34,35^

In the context of AMAs, a range of empirical modeling techniques has been utilized, drawing upon variables such as transformation temperatures,^36^ atomic dimensions,^37^ valence electron distributions,^38^ and thermodynamic properties.^39−41^ These methodologies have provided valuable insights into the conditions that are conducive to metallic glass formation. Recent advancements in ML have facilitated the successful prediction of a diverse array of properties in AMA materials, including the glass-forming ability of binary metallic alloys,^42^ the transformation temperatures of shape memory alloys,^43^ among other attributes.^31,44−47^ The outcomes of these investigations affirm the efficiency of ML methodologies in identifying novel metallic glasses and accurately predicting their characteristics. Nevertheless, most of this research has been constrained to specific compositions of metallic glasses, and there remains a gap in developing a comprehensive modeling framework that is capable of predicting the glass-forming ability of novel alloys.

Additional challenges exist among the ML methods. For instance, the main difficulty encountered in applying graph representation learning to HEAs lies in their characteristically simple lattice structures. The use of neighborhood graphs to represent HEAs proves to be inefficient, as such representations capture merely a specific instance of the underlying stochastic configurations, which fails to generalize the inherent randomness effectively. Moreover, there is a noticeable shortfall in initiatives that are directed toward enhancing model interpretability in relation to the prediction of material properties.

It is also important to examine the various approaches to feature selection that are present in the scientific literature. In the field of materials informatics, various feature selection methods exist and they are pivotal for improving the efficacy and interpretability of predictive models. Among these methods, filter methods based on correlation coefficients are particularly prevalent. Typically, these involve constructing a correlation matrix using Pearson correlation coefficients to assess the correlation or interdependencies among exploratory features. Features that are highly correlated are subsequently eliminated based on a predefined threshold value, while those exhibiting a large correlation with the target variable are retained.^48^

Another key technique in feature selection is the wrapper method. This technique employs predictive models, such as linear and tree-based models, to iteratively determine the importance or relevance of features. During this process, features are either added or removed while continuously monitoring the model performance, using either a sequential or recursive approach. Moreover, the selection of an appropriate estimator is important, as it involves balancing model complexity against training costs.^49^ For instance, while linear models are simpler to train and interpret, they are limited in their ability to detect nonlinear relationships or interactions among variables.

Embedded methods represent another popular approach in feature selection, whereby the feature-selection process is integrated into the model training phase. Various methods exist such as Lasso and Ridge regression. These regularization techniques penalize the magnitude of the feature coefficients, which effectively facilitates feature selection.^50^ Meanwhile, tree-based methods have gained significant popularity. Techniques such as decision trees and ensemble methods, including random forests, inherently conduct feature selection by selecting the most informative features during the construction of the trees, although the specific process differs among algorithms.^51^ This approach enables the ranking of feature importance, thereby allowing the selection of the most salient features. However, caution is necessary when relying exclusively on this method, as high multicollinearity among features can obscure their true relevance by evenly distributing the feature-importance scores among highly correlated features.

Dimensionality reduction methods are also commonly employed, despite their challenges in interpretability. These techniques transform the feature space to reduce the number of features, while preserving most of the information in the original data. Methods include: (i) Principal Component Analysis,^52^ which reduces the feature space by transforming features into a set of linearly uncorrelated components, and (ii) t-Distributed Stochastic Neighbor Embedding,^53^ which focuses on reducing dimensions while maintaining the distances between data points. These methods enable the identification of important components or the generation of embeddings that can be used to train a predictive model.

Despite the proliferation of feature selection techniques, it is common to see studies in the scientific literature that rely on just one or two of these methods, or even none. Where they are employed, these methods are often implemented in an ad-hoc basis, with limited consideration being given to their limitations or the computational costs that they incur. Such practices can lead to suboptimal feature selection which adversely affect the performance and interpretability of ML models. Suboptimal feature selection can also result in substantial, unnecessary computational cost, which escalate with the increasing number of data points and exploratory features that are considered. It is important to recognize that different feature selection techniques have distinct advantages and limitations. For instance, filter methods are computationally efficient but often overlook interactions between features. Conversely, wrapper methods consider feature interactions. However, they can be prohibitively time-consuming and resource-intensive, particularly with data sets with high-dimensionality. Embedded methods provide a balanced approach but struggle to effectively manage highly correlated features. A distinct trade-off exists between the thoroughness of feature selection and the practicality of computational requirements.

The development and application of a fully systematic ML-based framework for feature analysis and engineering remain relatively underexplored areas. Although numerous feature selection methods exist, their sporadic use in the literature underscores the necessity for a more methodical strategy. Adopting a structured approach that integrates multiple feature selection techniques with statistical and information-theoretic analyses, as well as an effective hyperparameter tuning process, could offer a more balanced and comprehensive understanding of the importance or relevance of features in relation to the target material property. It is clear that such implementation can lead to a more robust, interpretable, and efficient modeling framework in materials informatics.

To this end, we herewith employ the gradient boosted and statistical feature selection (GBFS) workflow, which we have designed for materials-property predictions.^54^ The GBFS workflow integrates a distributed gradient boosting framework, in conjunction with exploratory data and statistical analyses and two-step multicollinearity treatments, to discern a subset of features that is highly relevant to the target variable or class within a complex feature space. This affords minimal feature redundancy and maximal relevance to the target variable or classes. The efficacy of the workflow has been showcased in various materials-property predictions.^22,54−56^ Here, we apply the GBFS workflow to predict properties of HEAs and AMAs based solely on their chemical compositions. This general-purpose ML framework has been tailored to address the shortcomings observed in the existing research domain of alloys. These limitations encompass a lack of model interpretability, in addition to the absence of a systematic approach for feature analysis and engineering.

## 2 Methods

### 2.1 Data Sources

**Figure 1:**
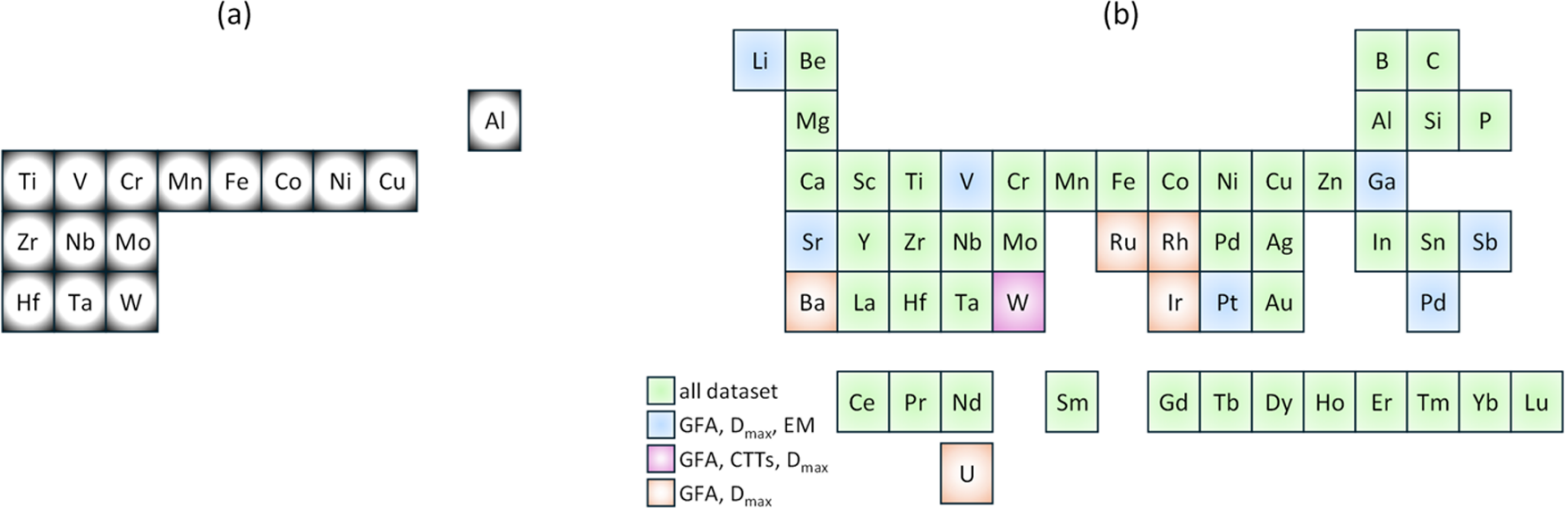
Periodic table highlighting the compositional space represented by the (a) HEA and (b) AMA data sets, where GFA stands for glass-forming ability, *D*_max_ the critical casting diameter, CTTs the characteristic transformation temperatures, and EM the elastic moduli.

One data set pertaining to HEAs was derived from the study conducted by Zhang et al.^31^ This data set comprises 7086 cubic quaternary HEA structures with crystal structure information and various properties, including the universal anisotropy, Zener anisotropy, Pugh ratio, elastic properties, formation enthalpy, total energy of structure, and Wigner–Seitz radius. The compositional space represented by the data set encompasses 14 chemical elements, as depicted in Figure 1a. Zhang et al. validated these data through comparative analysis against both experimental and computational findings reported in the existing literature.^57−66^ Consequently, this data set stands as the most extensive compilation of HEA structures, offering calculated information on stability and elastic properties.

Another data set comprises the mechanical properties and observed phases of HEAs compiled by Borg et al.^33^ This comprehensive database amalgamates 1545 records sourced from 265 scholarly articles, encompassing a diverse array of mechanical properties for 630 HEAs. It significantly extends a previously established database by integrating new data published since 2019, thereby enhancing the breadth and depth of available information for HEA research.

The data set used to study the AMA materials was compiled from an extensive array of sources, incorporating studies that span a significant breadth of the literature in this field.^67−79^ The data encapsulate variables, including the glass-forming ability of bulk metallic glasses (BMGs) with 6471 records, characteristic transformation temperatures (CTTs) with 674 records, critical casting diameter (*D*_max_) with 5934 records, and elastic moduli with 278 records. The data set for the glass-forming ability of BMGs and *D*_max_ contains 54 distinct chemical elements, while data sets related to CTTs and elastic moduli contain 42 and 48 chemical elements, respectively. The chemical elements comprising these data sets are illustrated in Figure 1b. This collective data set offers a comprehensive perspective on the attributes relevant to the study of metallic glass materials.

### 2.2 Feature Descriptors

For the construction of a high-dimensional feature vector, this study harnessed a suite of composition-based descriptors, leveraging the featurization capabilities of Matminer^80^ and Pymatgen.^81^ Subsequent feature augmentation involved the computation of statistical measures across elemental properties that are unique to each chemical entity. These computations were informed by a range of data repositories, encompassing Magpie^82^ and Pymatgen^81^ resources, as well as the predictive insights from the Deml^83^ database and the MatErials Graph Network^84^ (MEGNet) model, which utilizes neural-network embeddings for elemental characterization.

### 2.3 GBFS Workflow

**Figure 2:**
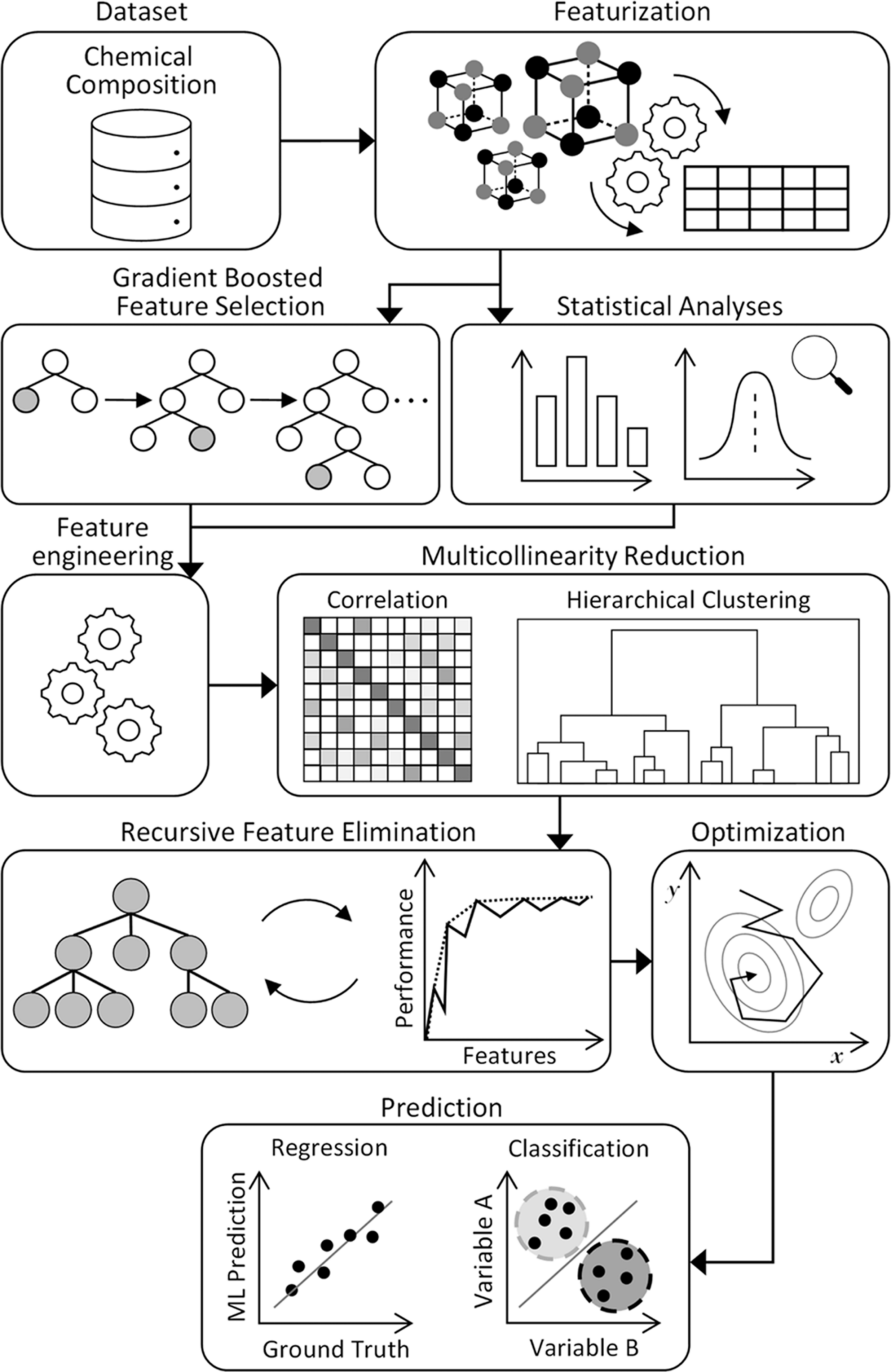
Overview of our operational workflow as described in Section 2. See ref (54) for a more detailed description. A portion of the figure has been reproduced with permission from ref (54).

The GBFS workflow presented in this study integrates several key elements: (i) the implementation of a gradient boosting framework tailored to identify a feature subset with maximum relevance to the target variable or class; (ii) the deployment of statistical analyses on preliminary features to identify those that are statistically significant to the target variable or class; (iii) a feature engineering phase to develop supplemental features; (iv) a dual-stage multicollinearity mitigation process that employs both correlation and hierarchical clustering analyses to minimize feature redundancy; (v) the application of recursive feature elimination (RFE) for feature refinement; and (vi) utilization of Bayesian optimization for configuring the architecture of the ultimate ML model. The schematic representation of the workflow is illustrated in Figure 2. While a comprehensive description of our GBFS workflow has been provided by Jung et al.,^54^ we herewith offer a concise summary of its key attributes to clarify how our approach distinguishes itself from the aforementioned studies.

Our modeling strategy presents considerable advantages over previous work by being highly systematic and, therefore, reducing the need for human intervention in both the feature selection and model development phases of its workflow. Initially, an extensive set of exploratory features is compiled. This is followed by calculations of the loss reduction or variance gain (i.e., feature relevance score) caused by each feature; the outcome of which provides a preliminary ranking of the features that is based on the derivatives (i.e., the gradients) of a loss function. Concurrently, a suite of statistical tests and analyses are carried out that are based on principles of probability theory and information theory. These processes efficiently identify the most relevant features for a given target variable or class. These features are subsequently used to generate additional features through a brute force method, which does not necessitate specialized domain knowledge. Nevertheless, manual intervention is available as an option to refine the feature engineering process. The most pertinent and statistically significant features, including newly engineered ones, are then evaluated for multicollinearity.

The approach to managing multicollinearity initially involves removing highly correlated features based on a predefined correlation threshold, which is followed by hierarchical clustering analysis to organize similar features into groups. A linkage threshold is established, enabling the algorithm to automatically select a representative feature from each cluster. The rationale for this selection process is that similar information or insights can be obtained from a single representative feature within each cluster, thus simplifying the feature space without sacrificing essential information. RFE is then performed using a greedy-based search algorithm that recursively removes features until the specified number of features is retained or no deterioration in the model performance is observed. Simultaneously, permutation-importance analysis is performed, which entails randomly rearranging the values of a single feature in order to assess its effect on a chosen set of performance metrics. This step helps to understand the influence of individual features on the predictive accuracy of the model.

This detailed process culminates in a systematically selected subset of features that are then used to perform Bayesian optimization. During this stage, the most effective model architecture is autonomously determined using only the training set, without requiring any human intervention throughout the process. After optimizing the final predictive model, it is assessed using the test set—this represents the first and sole use of this data set. This methodology ensures that our ML model is both robust and effective, trained within a feature space that is meticulously refined and selected by the algorithms, thereby eliminating human bias. Such a strategic approach ensures an optimal contribution of selected features to the predictive accuracy of our model, while effectively minimizing the impact of high correlations and redundancy among the input features. By greatly simplifying the feature space, this approach addresses potential overfitting issues and inherently incorporates regularization to achieve model generalization. This highlights the sophisticated and reliable nature of our highly systematic analytical approach. In the sections that follow, we will detail the results associated with each component of the GBFS workflow.

It is imperative that the sequence of stages within the proposed workflow is strategically designed to optimize a set of performance metrics while also minimizing the computational costs that are associated with the feature-selection process and subsequent model optimization. Here, we outline the rationale behind their sequencing. When a pair of highly correlated features is considered (e.g., above a correlation threshold of 0.8), they do not necessarily yield the same loss reduction or variance gain (i.e., feature relevance score) when growing the optimal tree during the initial stage of the GBFS workflow. That is to say that the first and second-order derivatives of the loss function will certainly vary between the two features. Likewise, feature rankings based on statistical significance testing may vary; for example, one feature may be identified as having a slightly stronger relative association with the target variable or class, even though the significance of its correlated counterpart could be similarly substantial. Consequently, we conduct the multicollinearity reductions after the initial GBFS stage and the statistical analyses, to prevent the premature elimination of features that may be more relevant to the target variable or class. Without this careful ordering, the multicollinearity treatment will arbitrarily discard correlated features based on a predefined correlation threshold, potentially ignoring the fact that one feature might possess more information about the target. This could lead to selecting a suboptimal subset of features for the final ML model. To prevent this issue, a thorough manual review of feature pairs would be necessary. However, such manual intervention would undermine the systematic nature of the feature-selection process that we aim to establish.

Furthermore, the initial GBFS stage eliminates the majority of exploratory features (≳90%) from being considered, thereby significantly lowering the computational costs that are associated with the subsequent downstream feature-selection or elimination processes. The task of mitigating multicollinearity to enhance model generalization and the risk of overfitting is considerably simplified at this point since the majority of exploratory features have already been excluded. Additionally, the outcomes can be readily visualized using diagrams such as dendrograms, given that we are dealing with such a refined subset of features. RFE then becomes the concluding step in our workflow, which aims to identify the smallest subset of features that maintains optimal model performance. During this phase, the least relevant features are systematically removed until the designated number of features is obtained or a chosen performance metric has stabilized. Since RFE follows a greedy-based search algorithm, it is computationally efficient to apply this method to the smallest subset of features before employing Bayesian optimization to determine the model architecture. For a more in-depth elucidation of our workflow, the reader is referred to the detailed methodology described by Jung et al.^54^

### 2.4 Evaluation Metrics

We examined the results of our regression analysis using the mean absolute error (MAE) and the mean squared error (MSE) as performance metrics, along with the coefficient of determination (*R*^2^), which is defined as the square of the Pearson correlation coefficient (*R*):
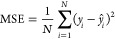
1
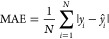
2
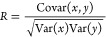
3where *y*_*i*_ represents the observed values, *ŷ*_*i*_ denotes the predicted values from the model, and *N* is the total number of observations. The covariance is given by
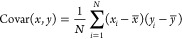
4with  and  representing the variance of the independent variable *x* and dependent variable *y*, where  and  are the mean values of *x* and *y*, respectively. The coefficient *R* ranges between −1 and 1, indicating the strength and direction of a linear relationship between the variables.

We evaluated the performance of our classification analysis within the GBFS workflow using the area-under-the-curve of the receiver-operating-characteristic curve (AUC-ROC), overall accuracy, and the F1-score. The F1-score is calculated as the harmonic mean between precision and recall, as delineated by the following expressions:
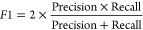
5
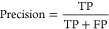
6
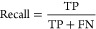
7
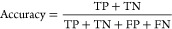
8where TP and TN are true positive and true negative, and FP and FN are false positive and false negative, respectively. Consistent with established literature, we employ these metrics to maintain uniformity in the evaluation across various regression or classification tasks.

## 3 Results and Discussion

### 3.1 High-Entropy Alloys

#### 3.1.1 Case Study One: Bulk Modulus Prediction

We begin by evaluating the efficacy of our GBFS workflow for HEAs in comparison to a variety of ML strategies that employed the aforementioned data set. Specifically, the comparative analysis focuses on the prediction of the bulk modulus (*K*) for HEAs, categorizing the alloys into equimolar and nonequimolar groups.

Table 1 compares the performance of various methods in the prediction of *K* for both the equimolar and nonequimolar compositions. We compared our GBFS results against those afforded using the following ML methods: Deep Sets,^85^ gradient boosting decision trees (GBDTs),^86^*k*-nearest neighbor (KNN),^87^ linear regression (LR),^88^ random forest (RF),^51^ and support vector machine (SVM).^89,90^ We observe lower MAEs in predicting *K* for equimolar quaternary HEAs in contrast to their nonequimolar counterparts, suggesting that property predictions pose greater challenges for the latter group. Among the evaluated predictive methodologies, LR and KNN recorded the highest MAEs, aligning with expectations that linear or simpler models would yield suboptimal performance. Conversely, SVM, RF, GBFS, and Deep Sets methods demonstrated enhanced predictive accuracy.

**Table 1:** MAE for the Prediction of Bulk Modulus (*K*) for Quaternary HEAs

	MAE (GPa)	
method	equimolar quaternary HEAs	nonequimolar quaternary HEAs
GBFS	4.196 ± 0.432	6.415 ± 1.071
Deep Sets	4.596 ± 0.639	6.025 ± 0.415
GBDTs	6.033 ± 0.349	8.622 ± 0.485
KNN	14.953 ± 0.620	12.508 ± 0.424
LR	7.891 ± 0.461	11.179 ± 0.414
RF	7.488 ± 0.574	9.607 ± 0.531
SVM	5.829 ± 0.536	7.636 ± 0.506

Notably, GBFS and Deep Sets methods significantly surpassed other ML-based approaches, with our GBFS workflow achieving the lowest MAE during 10-fold cross-validation for equimolar quaternary HEAs and the second lowest MAE for nonequimolar systems. More specifically, our GBFS approach demonstrated remarkable predictive accuracy for equimolar systems, recording an MAE of 4.196 ± 0.432 GPa, an root mean square error (RMSE) of 6.152 ± 0.865 GPa, and an *R*^2^ of 0.958 ± 0.012 in a 10-fold cross-validation. Similarly, for nonequimolar systems, our GBFS approach achieved an MAE of 6.415 ± 1.071 GPa, an RMSE of 9.781 ± 2.575 GPa, and an *R*^2^ of 0.914 ± 0.050. The apparent increase in the standard error in the latter case accounts for approximately 68% of the predictions in nonequimolar systems using the GBFS approach; this is attributable to the inclusion of 132 additional quaternary nonequimolar HEA compositions in the cross-validation process. These distinct compositions were not included in the original training and validation sets, which accounts for the reduced standard errors observed in the alternative models. This exclusion highlights the impact of data set diversity on model accuracy and error metrics.

**Figure 3:**
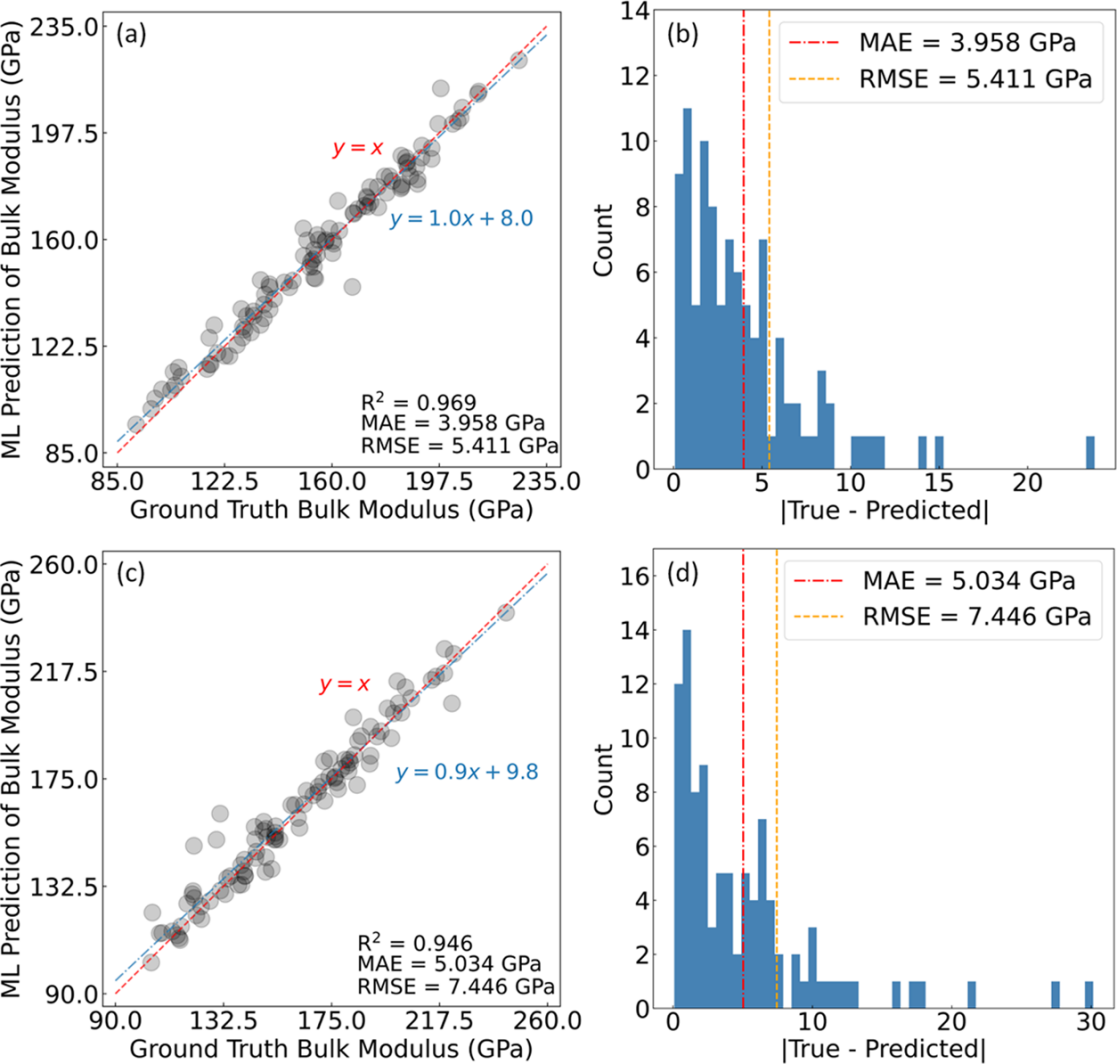
Regression of the ML-based predictions of HEA bulk modulus (*K*) against literature values in the out-of-sample test set and the corresponding distribution of absolute errors. The subfigures (a) and (b) are associated with equimolar quaternary HEAs, whereas (c) and (d) are associated with nonequimolar quaternary HEAs. The dashed red line is drawn to represent the hypothetical case, where the ML-based prediction would equal the literature values. The blue dot-dash line is a linear fit generated using the OLS method. Within the error distribution plots, the red dotted line symbolizes the MAE, and the orange dashed line denotes the RMSE.

The results obtained from the regression analyzes on the out-of-sample test set are depicted in Figure 3. The GBFS-based model demonstrated effective generalization across unseen chemical compositions within both equimolar and nonequimolar systems. In the case of equimolar compositions, the GBFS-based model attained an MAE of 3.958 GPa, RMSE of 5.411 GPa, and an *R*^2^ of 0.969. A linear regression, conducted via the Ordinary Least Squares (OLS) method, yielded a gradient of 1.0 and a *y*-intercept of 8.0, revealing a slight positive bias in the predictions for lower values of *K*. Nonetheless, this systemic bias is considered minimal within the observed range of *K* values for HEAs. The distribution of absolute errors further indicates that ca. 63% of predictions exhibit an error under 4 GPa, ca. 35% display an error under 2 GPa, and ca. 19% maintain an error below 1 GPa.

For the nonequimolar compositions, the predictive model achieved an MAE of 5.034 GPa, an RMSE of 7.446 GPa, and an *R*^2^ of 0.946 on the out-of-sample test set. A linear regression analysis resulted in a gradient of 0.9 and a *y*-intercept of 9.8, indicating a mild positive bias for predictions at lower *K* values and a slight underestimation for higher *K* values. Similar to the equimolar scenarios, this systemic bias is deemed relatively small within the range of *K* values considered. The distribution of absolute errors reveals that ca. 42% of predictions have an error below 4 GPa, ca. 25% are under 2 GPa, and ca. 10% fall below 1 GPa. Reflecting expectations, the nonequimolar compositions exhibit greater uncertainty, with a smaller proportion of predictions achieving errors that are below these thresholds in comparison to their equimolar counterparts. Please refer to Supporting Information 1 for details on the quantification of uncertainty.^91,92^

Extending this comparative analysis to encompass ML models reported in other studies presents challenges owing to the variation in the training sets. For instance, the AFLOW-ML and JARVIS-ML models reported MAEs of 8.68 and 10.5 GPa, respectively, in their predictions of the bulk modulus for inorganic structures.^93,94^ Moreover, our prior investigation, which focused on predicting the Voigt–Reuss–Hill approximation for the bulk modulus of isotropic polycrystalline materials, achieved an MAE of 1.167 GPa.^54^ This notably lower MAE is ascribed to the larger data set size and the integration of structural features in that study, while in the current analysis, our feature selection is strictly confined to attributes that stem from chemical composition, in addition to exclusively focusing on compositions that are relevant to the domain of HEAs.

#### 3.1.2 Gradient Boosted and Statistical Feature Selection Workflow

We now shift focus to the outcomes associated with each component of the GBFS workflow shown in Figure 2, as applied to our case study. The GBFS methodology was employed to distill a refined subset of 26 features from an initial pool of ca. 750 exploratory features and an additional 42 engineered features. The ensuing discussion outlines the results from this meticulous feature-selection process.

##### 3.1.2.1 The GBFS Process

**Figure 4:**
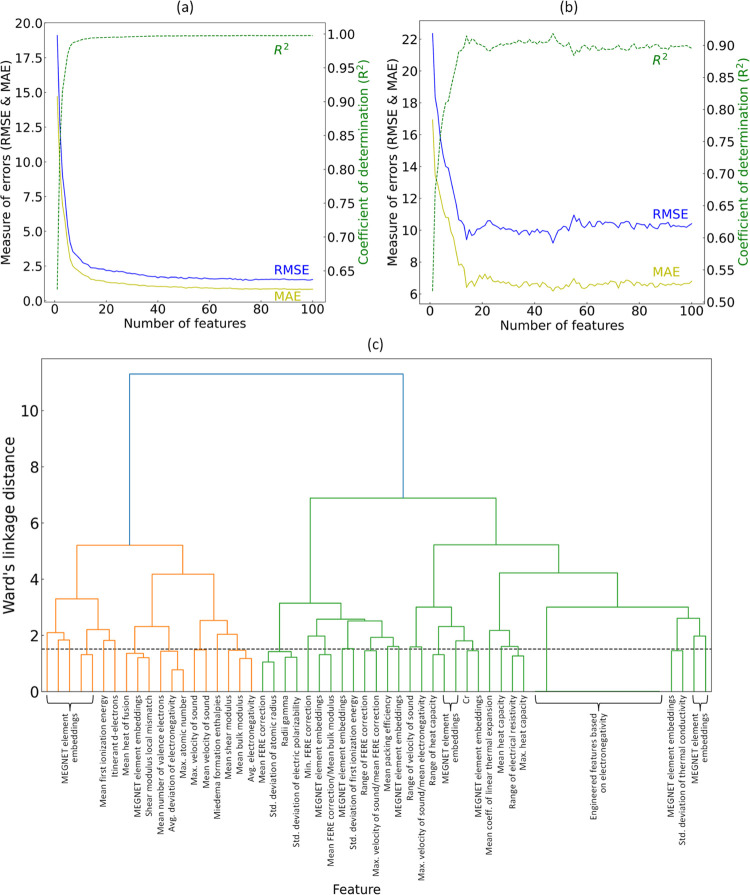
GBFS results for the prediction of *K* values in equimolar quaternary HEAs. The figure shows the performance of GBDTs on (a) the training set and (b) the validation set, where regression models are trained recursively with an increasing subset of features, beginning from the most relevant feature based on the realized total loss reduction. (c) Multicollinearity reduction for the regression analysis, showing the dendrogram of the hierarchical agglomerative clustering using the remaining 59 features after performing the correlation analysis. The dashed horizontal line in black represents the distance threshold of 1.5 unit of Ward’s linkage distance.

Initially, a recursive training of GBDTs with gradually expanding feature subsets was conditioned on the convergence of key performance indicators—MAE, RMSE, and *R*^2^—where the feature importance ranking was ascertained by the total loss reduction realized through the ML training process. The efficacy of regression models throughout this process, as assessed on both the training and validation sets, is illustrated in Figure 4. It was observed that, in both data sets, the performance metrics stabilized before the inclusion of ca. 50 features. They demonstrated slightly diminished effectiveness on the out-of-sample validation set as anticipated. Additionally, the convergence process on the validation set was marked by increased volatility, a predictable outcome considering that such a data set is out-of-sample and was randomly selected from the larger training set. At this juncture of the workflow, potential multicollinearity among exploratory features was not specifically addressed. The presence of such correlations can lead to a uniform distribution of total loss reduction across correlated features, thus obfuscating the genuine influence of individual features on the target variable.

##### 3.1.2.2 Feature Analyzes and Feature Engineering

Concurrently, we employed hypothesis-based testing methods of a bivariate form, focusing primarily on understanding the causal relationship between an exploratory feature and the target variable. For instance, a comparison of means was conducted using the F-test in one-way analysis of variance (ANOVA). This involves a correlation analysis using *R* for two continuous features, where the ANOVA approach to regression analysis is taken by converting *R* into a regression F-statistic. These hypothesis-based testing methods were employed for statistical inference, with the statistical significance of an exploratory feature being inferred from the test statistics that were generated by hypothesizing the existence of an association between two features.

Additionally, mutual information (MI) analysis was performed. The concept of MI was employed to quantify the level of the mutual dependence between two features, measuring the amount of information, or entropy, gained for a feature through the observation of another. For a pair of features, MI assesses the disparity between their joint distribution and the product of their marginal distributions, with a higher MI value indicating a greater mutual dependency between the two features or variables. We adopted an MI estimator based on entropy estimations derived from KNN distances.

The linear association of each continuous exploratory feature with the target variable was examined through a normalized F-statistic for relative comparison. This revealed that the feature demonstrating the highest linear association with the target variable is associated with the mean electronegativity of the elements present in the chemical composition with normalized F-statistics of 1.0, as estimated from reputable data sources such as Pymatgen^81^ and Deml.^83^ This is followed closely by the fitted elemental-phase reference energies (FERE) from Deml^83^ with normalized F-statistics of 0.65.

The MI analysis revealed that the greatest amount of entropy gain was realized with the estimate of mean value of *K* with a normalized MI score of 1.0. This specifically refers to the average *K* value of the elements within a particular chemical composition as sourced from Pymatgen.^81^ Following closely were features including the average electronegativity, the average shear modulus of the elements, the minimum coefficient of linear thermal expansion across the elements in the composition, and the average deviation in Goldschmidt’s atomic volume per atom within the chemical composition, each of which achieved a normalized MI score exceeding 0.72.

In essence, the MI analysis, incorporating the KNN method, suggests that more accurate predictions of *K* can be achieved by considering these features. It is noteworthy that the estimation of MI involves assessing the probability density distribution and marginal distributions of the two variables of interest. However, estimating these distributions becomes increasingly challenging when dealing with higher-dimensional data, given the limited number of samples with respect to the number of dimensions. This limitation often leads to substantial variations in probability and, as a result, the estimated information gain in MI analysis may suffer from the high dimensionality of the data set or an inadequate sample density with respect to the dimension of the feature space. The features identified through the GBFS process and statistical analyses were used to engineer new features via the brute-force method. This process resulted in an additional 42 features, leading to a total number of 117 features that formed the preliminary subset of features for the regression analysis.

##### 3.1.2.3 Multicollinearity Reduction, Permutation Analysis, and Recursive Feature Elimination

A subsequent phase of the GBFS workflow employed multicollinearity reduction within the data set. This assessed the permutation importance of the selected features, and conducted RFE to ascertain the final subset of features for Bayesian optimization of the final predictive ML model.

To mitigate the effects of multicollinearity in the data set, features with a correlation coefficient of 0.8 or higher were systematically removed, resulting in a reduced subset of 58 features. The next level of remediation for multicollinearity effects involved employing a hierarchical cluster analysis, using the Spearman rank-order correlation with a Ward’s linkage distance threshold of 1.5 units. This led to the retention of 31 features, as only one feature from each cluster was chosen. The optimal distance threshold was determined using the Elbow method. The corresponding dendrogram, shown in Figure 4c, depicts the hierarchical agglomerative clustering, with cluster formation as one ascends the dendrogram.

**Figure 5:**
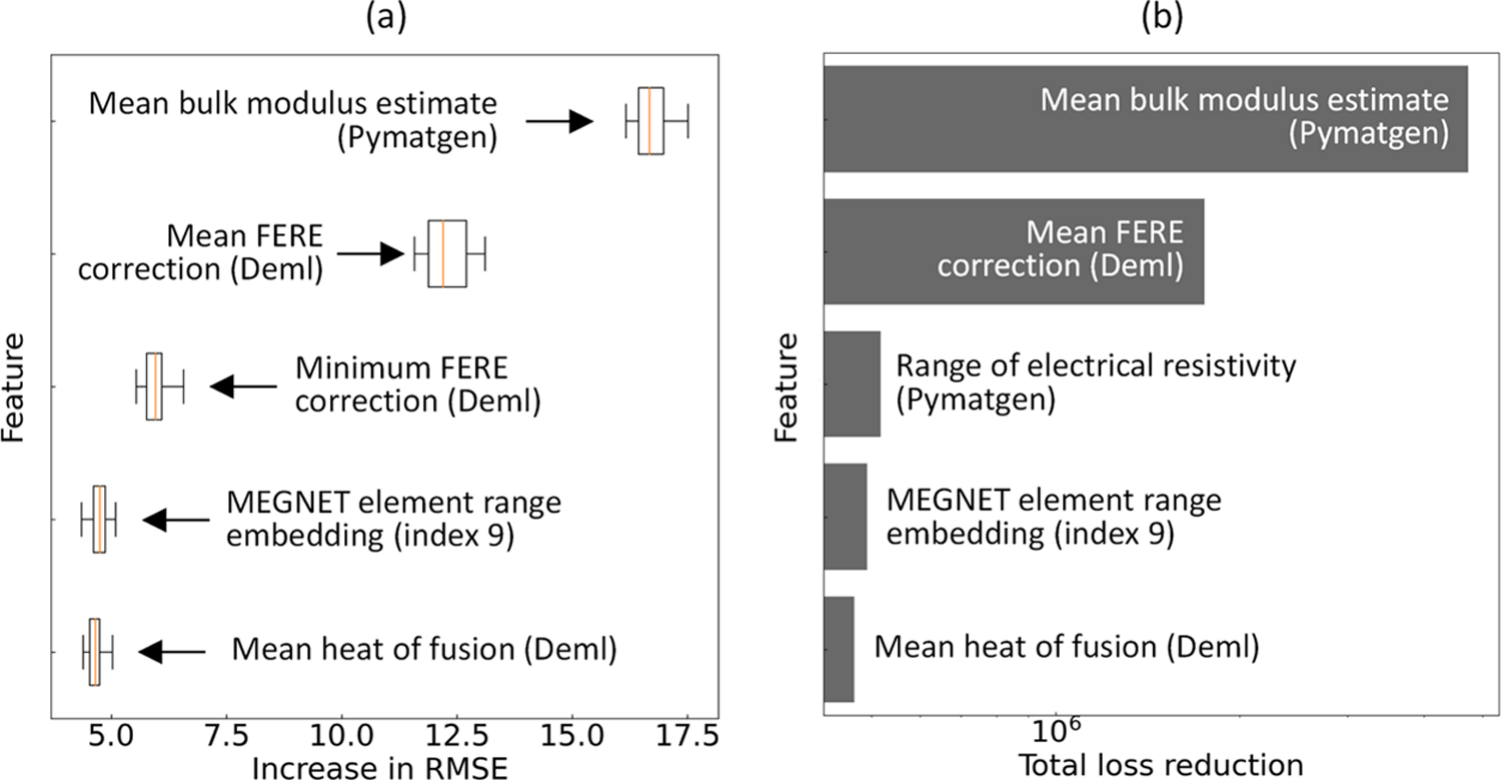
(a) Feature relevance and (b) permutation-based feature importance plots for the regression analysis of *K* in equimolar quaternary HEAs, displaying the five most significant features.

The results of the 10-fold permutation feature-importance analysis are shown in Figure 5a. Permutation feature importance is quantified as the reduction in a model performance when a single feature is randomly shuffled. This process disrupts the association between the feature and the target, making the decrease in the model performance indicative of the model’s reliance on that particular feature. The 10-fold feature permutation analysis indicates that the most important feature is the mean *K* value of the elements within a given chemical composition from Pymatgen.^81^ This is followed by the mean and minimum FERE from Deml,^83^ MEGNet element embedding,^84^ and the mean heat of fusion for the elements within a given chemical composition, listed in the order of permutation importance. The results are consistent with the statistical analyses that were conducted independently.

It is important to highlight that element embeddings derived from graph-neural-network models, exemplified by MEGNet, encapsulate chemical periodicity and trends that are inherent within the periodic table. While the interpretation of these individual embeddings may present challenges, their utility has been demonstrated in transfer-learning scenarios. Specifically, embeddings can be extrapolated from a material-property model that has been trained on a comprehensive data set to enhance the predictive performance of models based on more limited data sets. In this case study, we leveraged such learned embeddings to refine the prediction accuracy of *K* for equimolar quaternary HEAs.

Following the aforementioned analysis, the optimal subset of features was determined by eliminating further features through 10-fold RFE, employing negative RMSE as the performance metric. This process led to the identification of the final subset comprising 26 features, which were chosen from an initial pool of ca. 750 original features and 42 engineered features. These 26 features bear the highest relevance to the target variable without any prior knowledge of the domain.

##### 3.1.2.4 Model Optimization & SHAP Analysis

A two-step optimization process was undertaken to determine the final predictive model. The hyperparameters of the model were optimized using a combination of grid search and Bayesian optimization. An initial hyperparameter tuning process was performed by scanning the hyperparameter space with the grid-search method. This subsequently identified the region in which Bayesian optimization should be applied. Bayesian optimization proves to be particularly effective for an objective function that has no closed form, is expensive to evaluate and is well suited to problems whose evaluations result in noisy responses. Figure 5b displays the five features that achieved the most significant total loss reduction in predicting the target variable by the final regression model. The features identified are largely in agreement with those highlighted in the permutation analysis, with a notable exception being the inclusion of the range of electrical resistivity among the elements within a specified chemical composition, as sourced from Pymatgen.^81^

**Figure 6:**
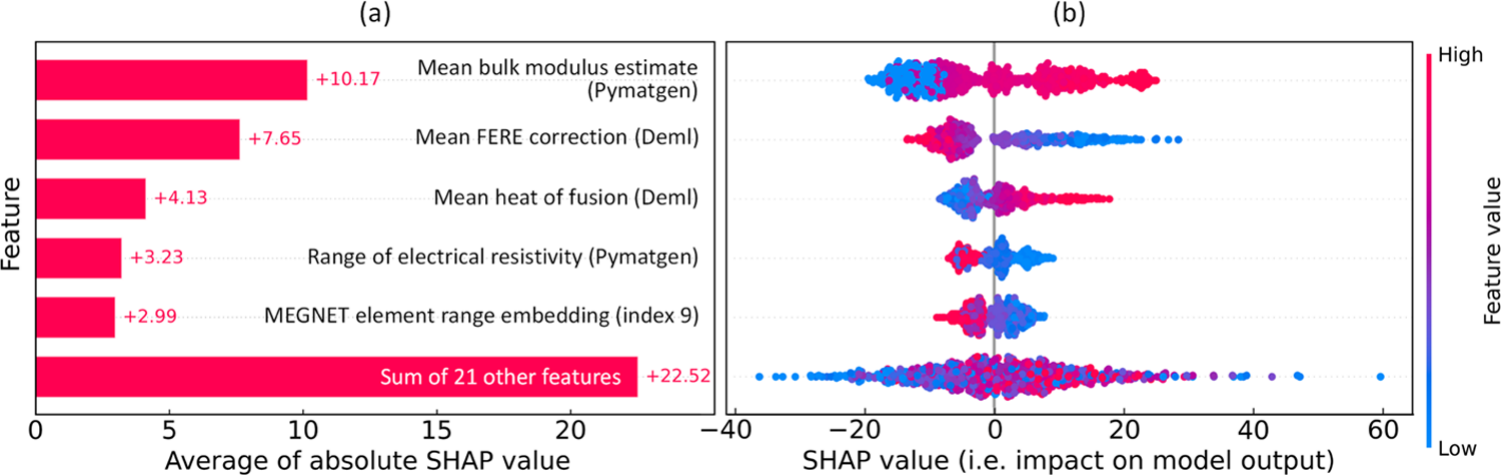
Results based on the SHAP framework: (a) the average contribution (i.e., the mean absolute SHAP value) of five features that are identified as having the greatest contributions to the model output. A positive SHAP value indicates a positive contribution to the prediction of *K* values. (b) The beeswarm plot illustrates the impact of these features on the model output by plotting each instance as a single data point together with the SHAP value on the *x*-axis, where the *y*-axis is consistent with (a). The color scheme corresponds to the original feature value and the broadening shows the density of instances (cf. the density plot).

An independent feature analysis was conducted using the SHapley Additive exPlanations (SHAP) framework,^95^ which is a game theoretical approach that explains the output of an ML model. This independent step was conducted solely to ensure that there are no significant discrepancies in our methodology; the SHAP-based analysis was not used to determine or influence the final subset of features in this study. Figure 6a displays the average contribution plot (i.e., the mean absolute SHAP value) of the five features identified as having the most significant contributions to the model output. The accompanying beeswarm plot in Figure 6b illustrates the impact of these features on the model output by plotting each instance as a single data point together with the SHAP value on the *x*-axis. These findings align with the features identified by the GBFS workflow (see Figure 5b), providing additional validation for the effectiveness of our modeling approach. The findings indicate that elevated values of the mean elemental *K* estimate and a larger mean heats of fusion correlate with higher *K* values in HEAs. Conversely, a greater mean FERE correction and a wider range of elemental electrical resistivity are linked to lower *K* values in HEAs.

#### 3.1.3 Feature Interpretation

Figure 5b details the five most influential features in our regression analysis of the bulk modulus. We now seek to rationalize their significant role. The most salient feature, as denoted by the realized total loss reduction, pertains to the mean bulk modulus estimate that is derived from the constituent chemical elements of an alloy. The identification of this feature having the highest relevance was anticipated, considering its direct material association with the measure of intrinsic elasticity or the resistance of a material to bulk compression. Insights from the beeswarm plot in Figure 6b reveal that higher values of this feature generally align with more positive SHAP values, corroborating our expectations regarding its predictive significance.

The prominence of the mean FERE correction across the constituent elements of an alloy as the second salient feature requires further explanation. The FERE model is used in computational materials science to modify or adjust the reference state energies of chemical elements to better align with experimental values. This correction helps improve the accuracy of thermodynamic models by providing more realistic assessments of formation energies in alloys.^96^ For instance, in the context of predicting properties such as the bulk modulus, the accuracy of elemental energies is essential. Incorporating the mean FERE correction as a feature ensures that the underlying thermodynamic calculations for predicting mechanical properties are anchored to well-adjusted, realistic energy states. This leads to more accurate predictions of the bulk modulus. The use of FERE-corrected energy values therefore enhances the reliability of these assessments, ensuring that the materials will behave as expected under mechanical stresses.

The range of electrical resistivity among constituent elements was identified as the third most relevant feature. The connection between electrical resistivity and the bulk modulus, though not immediately apparent, holds considerable importance for several reasons. Electrical resistivity offers insights into the electronic structure and bonding characteristics of a material. Materials characterized by strong metallic bonding typically exhibit lower resistivity owing to the unimpeded flow of electrons. This type of bonding directly impacts mechanical properties, such as the bulk modulus, since stronger bonds usually correlate with a stiffer material, thereby enhancing the bulk modulus. Therefore, variations in resistivity can serve as indirect indicators of changes in the bulk modulus. Moreover, high resistivity in metals and alloys is often linked to an increased presence of defects or impurities, which can adversely affect mechanical properties. These defects and impurities disrupt the regular atomic arrangement of a material structure and compromise its ability to compress uniformly under stress, which can result in a reduced bulk modulus.

We have previously discussed the challenges in interpreting MEGNet element embeddings. To gain clarity on how feature selection is influenced when these embeddings are omitted, we employed our GBFS workflow to train an alternative ML model. This analysis led to the identification of the average electronegativity among the constituent elements as one of the top five most relevant features. Electronegativity differences among atoms within a material are critical in determining the nature of bonding—ionic, covalent, or metallic. This, in turn, significantly impacts the mechanical properties of materials. For instance, materials characterized by ionic or covalent bonding, which involve atoms with greater electronegativity differences, tend to have higher bulk moduli because the strongly directional nature of these bonds effectively resist compression. Conversely, materials whose neighboring atoms exhibit similar electronegativity usually form metallic bonds. These materials display varying bulk moduli in line with the density of the electron clouds and the nature of bonding involved. Such variations in bonding characteristics directly affect how internal stresses are distributed when external forces are applied to a material, thereby influencing the material’s response to compression and its overall level of mechanical stability.

#### 3.1.4 Predicting a Wide Range of HEA Properties

With the foundations of our GBFS approach assured through its demonstrational efficacy in predicting *K* values, we sought to predict other properties for HEAs. These properties were derived from the cubic elastic constants through mathematical operations. For instance, the arithmetic Hill average for calculating the polycrystalline shear modulus (*G*) is given by
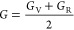
9where *G*_R_ and *G*_V_ represent the Reuss and Voigt shear modulus bounds, respectively. These bounds are expressed as
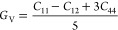
10
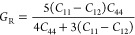
11with *C*_11_, *C*_12_, and *C*_44_ being the cubic elastic constants. Additional mechanical properties such as Young’s modulus (*E*), Poisson’s ratio (*v*), the Zener anisotropy ratio (*A*_z_), and Pugh’s ratio (*k*) are derived through the following equations:
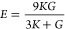
12
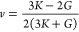
13
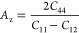
14
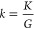
15noting that the crystal lattice is considered elastically isotropic when *A*_z_ = 1 and, in the context of cubic polycrystalline structures, the Voigt and Reuss bounds for *K* converge to the same value;^97^ thus, *K* = *K*_V_ = *K*_R_.

**Figure 7:**
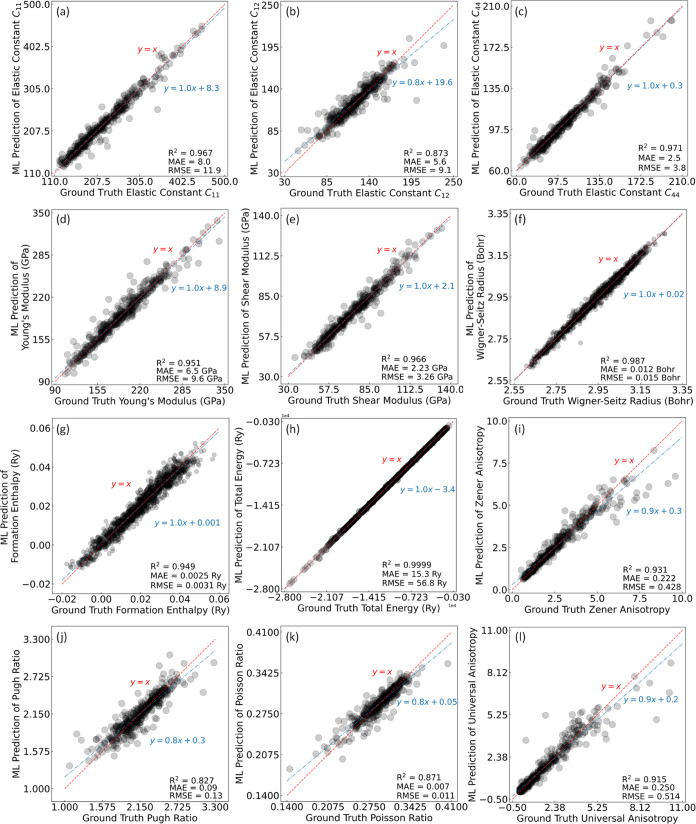
Property predictions for HEAs. The dashed red line is drawn to represent the hypothetical case, where the ML-based prediction would equal the literature values. The blue dot-dash lines represent linear fits generated using the OLS method, applied to various materials properties: (a) elastic constant *C*_11_, (b) elastic constant *C*_12_, (c) elastic constant *C*_44_, (d) Young’s modulus, (e) shear modulus, (f) Wigner–Seitz radius, (g) formation enthalpy, (h) total energy, (i) Zener anisotropy, (j) Pugh ratio, (k) Poisson ratio, and (l) universal anisotropy.

The additional properties examined in this study were sourced from the data set described in Section 2.1, which were created using high-throughput Density Functional Theory (DFT) calculations. These calculations were conducted using exact muffin-tin orbitals and coherent potential approximation (EMTO–CPA) methodologies.^31,98,99^ Our analyses exclusively employed features derived from chemical composition, in the same fashion as our predictions of *K*. For each target property, we processed the exploratory features through the GBFS workflow. This process facilitated the identification and selection of a distinct subset of features, which ultimately culminated in the development of a dedicated predictive model for each property evaluated. The regression outcomes on the out-of-sample test set are depicted in Figure 7, while the associated error distributions are presented in Supporting Information 2. The result pertaining to the classification of HEAs based on their experimentally observed structural symmetries is presented in Table 2, where the microstructure is classified into face-centered cubic (FCC), body-centered cubic (BCC) or other atomic arrangements. The corresponding confusion matrix can be found in Supporting Information 3.

**Table 2:** Summary of the Performance Metrics for the Classification of HEAs by Their Structural Symmetries

	precision	recall	F1-score
BCC	0.913	0.894	0.903
FCC	0.872	0.774	0.820
other	0.871	0.914	0.892
macro average	0.885	0.860	0.872
weighted average	0.884	0.883	0.883

The GBFS workflow evidence its broad utility in predicting 13 diverse properties of HEAs based solely on features derived from their chemical composition (Figure 7). The comparison between GBFS-based predictions of these properties and values reported in the literature demonstrates a high degree of agreement, with the minimum *R*^2^ value being 0.827 for the prediction of the Pugh ratio. Furthermore, the distribution of absolute errors (in Supporting Information 2) predominantly aligns toward the lower spectrum of the error scale, with a subsequent reduction in the error counts at higher values. In the case of classifying HEAs based on their experimentally observed structural symmetries, we obtained an AUC-ROC of 0.97, an F1-score of 0.88, and an accuracy of 0.88. These observations underscore the predictive precision of our GBFS workflow.

Additionally, it is helpful to highlight the variance observed between the directly predicted *K* values and those deduced from the predicted elastic constants. Zhang et al.^31^ observed that the maximum discrepancy between these approaches was about 8%, a level of variance that is deemed permissible considering the intrinsic uncertainties in DFT-calculated elastic constants, which can diverge from experimental measurements by up to ±15%.^100^ Accordingly, this substantiates the application of the mathematical operations to approximate material properties using eqs 9–15, which are formulated based on the predicted cubic elastic constants. A verification of the predicted elastic constants through the GBFS workflow confirms the mechanical stability criteria, as evidenced by observing the following criteria: *C*_11_ > 0, *C*_44_ > 0, *C*_11_ – *C*_12_ > 0, and *C*_11_ + 2*C*_12_ > 0. These findings are consistent with the criteria established by the EMTO–CPA methodology.

We now examine the variance among the prediction error. This examination is crucial, as analyzing key statistical measures provides only a partial view. A more detailed analysis of the error distribution, especially in the presence of heavy-tailed distributions, can offer deeper insights into the nature and accuracy of these predictions. Consequently, we have conducted a comparative analysis of the error distributions across five different ML methods, four of which relate to the study by Zhang et al.^31^ The five ML methods evaluated include: RF, KNN, GBDTs, Deep Sets, and GBFS. The set of boxplots in Supporting Information 4 illustrates the distributed deviations of predicted values from the true values for four HEA properties, namely bulk modulus (*K*) and the three elastic constants (*C*_11_, *C*_12_, and *C*_44_).

The predictive accuracy of *K* exhibits a narrow distribution around the zero line, with the first quartile (Q1) at −4.1 GPa, the median at −0.4 GPa, and the third quartile (Q3) at 2.8 GPa. The lower and upper whiskers terminate at −14.2 and 13.0 GPa, respectively, with the whiskers defined as 1.5 times the interquartile range (IQR) of 6.8. These results indicate that the predictions generally approximate the true values with minimal error. Notably, there are outliers that are located on either sides of the zero line, positioned outside the bounds defined by the whiskers. These three outliers signify notable deviations from the true values. Nevertheless, the majority of data points cluster near the median, underscoring a consistent predictive accuracy.

In comparison to other models such as GBDTs, RF and KNN, the variance in the prediction error from our GBFS-based model is considerably lower, evidenced by the significantly narrower region between the whisker ends. However, when compared with results from Deep Sets methodology, the latter appears to exhibit a marginally lower variance, which contrasts with other test-set results reported in this study. Zhang et al.^31^ attribute these enhanced results for the unseen HEAs, relative to their test results, to an averaging procedure that is applied across ten models. Their strategy effectively leverages an ensemble modeling approach, which contrasts with our single-model approach. Therefore, it can be inferred that while our GBFS workflow surpasses all the aforementioned methods discussed under a single-model framework, the adoption of an ensemble modeling strategy can further enhance predictive accuracy, as evidenced by the ensemble implementation of Deep Sets. Additionally, it is noteworthy that the Deep Sets approach generated a greater number of outliers, with the most extreme outlier exceeding 50 GPa, whereas the GBFS-based model recorded its furthest outlier at 24.7 GPa. This observation underscores differences in the robustness and stability of the predictive models under evaluation.

A consistent pattern emerges from an examination of the results for the three elastic constants. The error distributions for the GBFS-based models exhibit markedly lower variance, as indicated by a narrower boxplot when compared to those of the GBDTs, RF, and KNN models, particularly in the regions defined by the lower and upper whiskers. This finding corroborates our observation that the GBFS workflow provides a more stable model prediction compared to these models. Once again, the results from the ensemble Deep Sets demonstrate a lower variance in their prediction error. However, it is crucial to highlight that Zhang et al.^31^ constructed their boxplot using 132 quaternary HEAs for their unseen HEA data, while our analysis included around 400 unseen chemical compositions, representing a broader and more diverse set of data. This greater diversity in our data set is likely to be a significant contributory factor to the observed discrepancies in this comparison. Additionally, we reiterate that the prediction accuracy could be further enhanced by adopting an ensemble modeling approach, as demonstrated by the ensemble implementation of Deep Sets. These results support the notion that systematic feature analysis and selection, in conjunction with Bayesian optimization, can significantly enhance model prediction across a variety of material properties.

The statistical parameters for the error distributions of the elastic constants are delineated as follows. For *C*_11_, Q1 is −6.6, the median is −1.6, and Q3 is 4.4, with an IQR of 11.0. For *C*_12_, Q1 is −3.7, the median is −0.2, and Q3 is 2.8, with an IQR of 6.4. For *C*_44_, Q1 is −1.3, the median is 0.3, and Q3 is 2.2, with an IQR of 3.5. The predictions for *C*_11_ show a relatively wider spread compared to the other elastic constants, reflecting increased variability in the prediction errors. A relatively larger number of outliers, predominantly on the positive side, indicates a tendency of the model to underestimate *C*_11_ values at the extremes. In contrast, *C*_12_ displays a more compact distribution around the zero line, albeit with a slight leftward deviation in some data points, suggesting a marginal tendency toward overestimation. While *C*_12_ exhibits fewer outliers than *C*_11_, the presence of some outliers suggests the occasional production of substantial errors. The error distribution for *C*_44_ is the most contained among the elastic constants, with a symmetric boxplot around the zero line, indicating a balanced occurrence of overestimations and underestimations. Outliers are present at both extremes but are less pronounced than those for *C*_11_, implying that while errors do occur, they are generally less severe. Overall, the analysis indicates that prediction errors for *C*_11_ tend to be larger than those for other properties, possibly due to challenges in accurately modeling this elastic constant, which may be particularly sensitive to specific nuances in chemical composition of the alloys.

The analysis of chemical compositions identified as outliers for the properties of *K*, *C*_11_, *C*_12_, and *C*_44_ offers valuable insights into potential patterns or specific elements that may be contributing to deviations from expected values. The initial observation is that the majority of these compositions pertain to quaternary nonequimolar HEAs. This high level of elemental diversity and complexity could lead to greater variability in material properties, influenced by differences in atomic sizes, bonding characteristics, and electronic structures, which are critical in determining mechanical properties. Additionally, the prediction of these properties is based solely on features derived from their chemical compositions, making such greater variability expected.

In examining the presence of specific elements within the outlier chemical compositions, it is notable that Tungsten (W) and Hafnium (Hf) frequently occur. These heavy transition metals are likely to influence the mechanical properties of the alloys, possibly due to their high density and strong bonding characteristics. Additionally, Aluminum (Al) is another element commonly found in these outlier chemical compositions, which is known for its lightweight nature and corrosion resistance. However, its presence in high proportions might be unpredictably influencing the elastic properties, especially when combined with heavier or more brittle elements. These observations suggest that the inclusion of such metals could be pivotal in dictating the mechanical behavior of the alloys.

In the context of predicting the *K* values, the presence of several outliers, particularly those with high W content, suggests that *K* is sensitive to the incorporation of heavy metals. This is potentially due to their inherently high modulus of elasticity. Nevertheless, the outliers encompass a variety of alloying elements, including chromium (Cr), niobium (Nb), titanium (Ti), cobalt (Co), copper (Cu), and manganese (Mn). This high level of elemental diversity indicates that there is no single dominant pattern affecting the bulk modulus; rather, it appears that the combined interactions of multiple elements within a given alloy will significantly influence its mechanical properties.

In the analysis of elastic constants, a notable level of diversity in transition-metal composition is observed. Many outliers are characterized by a combination of transition metals, such as chromium (Cr), manganese (Mn), cobalt (Co), and nickel (Ni), which are recognized for their contribution to strengthening mechanisms but may also introduce greater variability in directional elastic properties, such as *C*_11_, *C*_12_, and *C*_44_. Moreover, the presence of minor percentages of elements, such as zirconium (Zr), hafnium (Hf), and vanadium(V) in certain alloys, correlates with these outliers, indicating that even trace components can profoundly influence elastic constants through their effects on crystal structure and bonding. In particular, the predictions of *C*_11_ and *C*_44_ show heightened sensitivity to combinations of Al with transition metals or heavier elements, reflecting the susceptibility of these elastic constants to lattice distortions or variations in atomic packing. Conversely, *C*_12_ displays sensitivity to a variety of elemental mixtures, without a predominant single-element influence, suggesting that the overall alloying strategy and the ratios of elements are critical.

It is evident that the presence of heavy transition metals in complex multicomponent systems markedly influences mechanical properties, with elements such as W, Hf, and Al playing crucial roles. The observed variability in properties such as elastic modulus and elastic constants across these alloys can likely be attributed to the intricate interactions among these elements within the alloy’s crystal structure, potentially impacting bonding characteristics and electronic interactions. This level of complexity underscores the need for a nuanced understanding of how elemental composition of advanced alloys affects their mechanical behavior.

Upon extending our examination to include additional properties that are depicted in Figure 7, we identified consistent chemical profiles and trends among the outliers in the prediction of various properties including Young’s modulus, total energy, Zener anisotropy, Pugh ratio, Poisson ratio, and universal anisotropy. We noted a prevalent use of Al and a combination of heavy transition metals, rare earth elements, or refractory metals such as Hf, Zr, Ti, Nb, W, and V. The majority of these outliers were linked to nonequimolar HEAs.

Further discussion is necessary to better understand the model predictions of various properties by concentrating on specific regions of interest relevant to different applications. This approach is essential because an accurate representation of the model performance can be observed by focusing on a region of particular interest for a given property. Analyzing whether or not predictions within this defined interval exhibit lower prediction errors will provide deeper insights into the effectiveness our ML models. Generally, the performance metrics that were calculated (i.e., *R*^2^, MAE, and RMSE) reliably reflect the accuracy of our models across their entire respective ranges, demonstrating minimal or consistent deviations from the actual values for target properties such as elastic constants, Young’s modulus, shear modulus, Wigner–Seitz radius, formation enthalpy, and total energy. Nevertheless, this consistency does not extend to properties such as Zener anisotropy, universal anisotropy, Pugh ratio, and Poisson ratio. Therefore, it is crucial to engage in further discussion regarding these properties to better understand the efficacy of our modeling approach.

Specifically, the Zener anisotropy or ratio, which is a dimensionless number used to quantify anisotropy in cubic crystals, indicates the extent to which a material deviates from isotropic behavior, with a value of 1 representing perfect isotropy. For HEAs, a Zener ratio close to 1 is therefore desirable. This ratio signifies isotropic behavior, indicating that the properties of a material will exhibits uniformity in all directions. A Zener ratio of 1 implies that the alloy will maintain consistent mechanical properties irrespective of the direction in which stress is applied, making it ideal for applications that demand uniform behavior across various orientations. In Figure 7, a noticeable deviation between the ground truth and the predictions is evident for Zener anisotropy values greater than 5, which is attributable to the scarcity of materials within this range. As a result, the accuracy of our model decreases when designing materials with high anisotropy. Nonetheless, since the majority of materials fall within the desirable isotropic value of 1, the deviations observed at higher values do not significantly undermine the overall effectiveness of this predictive exercise.

The Zener anisotropy or ratio is applicable only to cubic crystals. To address this limitation, the universal anisotropy index was formulated using variational principles of elasticity and tensor algebra, enabling the quantification of anisotropy for elastic crystals across all classes. For HEAs, a desirable universal anisotropy value is close to zero. A value of zero indicates isotropic behavior, meaning that a material has uniform elastic properties in all directions, which is typically preferred for various applications, as previously mentioned. Similar to the Zener anisotropy, the prediction performance shows noticeable deviations from the ground truth for universal anisotropy values approximately beyond 3. This is expected given that the majority of data points fall below this range. Nevertheless, since the desirable value is zero, the deviations observed at higher values do not substantially impact the overall efficacy of this predictive exercise.

The Poisson ratio is defined as the ratio of transverse strain to axial strain when a material is subjected to stretching or compression. It offers important insights into the ability of a material to undergo dimensional changes that are perpendicular to the load direction. A higher Poisson ratio, nearing the theoretical limit of 0.5 for isotropic materials, signifies greater ductility, allowing the material to stretch or compress with minimal volumetric changes. For HEAs, the optimal Poisson ratio typically falls between 0.3 and 0.35. This range is preferred in engineering applications due to its balance between ductility and volume conservation during deformation. In HEAs, maintaining a Poisson ratio within this specified range is considered to be ideal, as it enables the alloys to withstand mechanical stresses without undue deformation or failure. Once again, we observe that large deviations occur outside the targeted range, especially below a value of approximately 0.275.

The Pugh ratio, defined as the ratio of the bulk modulus to the shear modulus, demonstrates that a value greater than 1.75 indicates increased ductility. This characteristic is generally beneficial for applications that require materials to withstand substantial deformation without fracturing. Although there is no strict upper limit to the Pugh ratio that categorically defines it as undesirable, the suitability of a higher or lower Pugh ratio greatly depends on the specific application and required performance characteristic of a given alloy. While Pugh ratios significantly above 1.75 are indicative of enhanced ductility, they may also lead to reduced strength and hardness. Therefore, in situations where greater strength and hardness are essential, an exceedingly high Pugh ratio may not be advantageous. That said, similarly to the other properties discussed, we observe that relatively lower errors occur within the range of values that are typically considered to be suitable for HEAs.

#### 3.1.5 Blind Test on Quinary HEA Composition

The aforementioned predictions of various material properties in comparison with values reported in the literature demonstrate notable statistical merit. However, the validation of these results extends beyond mere statistical performance; it enables an evaluation against specific, well-characterized, HEA materials. We now showcase this model capability by performing a blind test study on the extensively studied quinary HEA system NiCoFeCrAl_*x*_,^101^ whose compositional range was previously unseen by the model; i.e., it is an out-of-sample test case.

This analysis emphasizes the prediction of *K* values and the Wigner–Seitz (W–S) radius, with the outcomes being presented in Table 3 together with the comparable results from the Deep Sets ML-based prediction and DFT-calculated values that employed the EMTO–CPA methodology. The outcomes demonstrate a high level of agreement, which is particularly notable given that the GBFS-based model received no training on quinary HEA compositions. An auxiliary analysis of mean absolute deviation reveals that the GBFS workflow exhibits lower variance with respect to literature values when compared to the Deep Sets ML-based model. Specifically, regarding the prediction of *K* values, the mean absolute deviation is lower by ca. 46%, and for the W–S radius, this deviation is observed to be an order of magnitude lower between the two ML methodologies.

**Table 3:** Predicted Wigner–Seitz (W–S) Radius and Bulk Modulus (*K*) for the NiCoFeCrAl_*x*_ Quinary System, as Obtained through the GBFS Workflow, are Juxtaposed with Findings Derived from Deep Sets and EMTO–CPA Calculations for the Preferred Structural Symmetry

	*K* (GPa)			W–S radius (Bohr)			
*x*	GBFS	deep sets	EMTO–CPA	GBFS	deep sets	EMTO–CPA	symmetry
0.1	198	199	200	2.622	2.620	2.611	FCC
0.25	197	195	197	2.627	2.628	2.619	FCC
0.3	197	194	196	2.633	2.631	2.622	FCC
0.375	194	192	194	2.634	2.635	2.626	FCC
0.5	188	189	190	2.637	2.641	2.632	FCC
1.25	171	166	171	2.671	2.680	2.667	BCC
1.3	170	166	170	2.673	2.678	2.670	BCC
1.5	163	161	167	2.678	2.689	2.675	BCC
2	159	151	159	2.692	2.706	2.690	BCC
2.5	144	145	153	2.711	2.721	2.701	BCC
mean absolute deviation	1.8	3.9		0.0065	0.0116		

### 3.2 Amorphous Metallic Alloys

Having demonstrated the utility of our GBFS workflow on predicting HEA properties, we now direct our attention to the analogous application of GBFS to another type of alloy, AMAs. Thereby, we examine their glass-forming ability, treating it as a classification problem, as opposed to the regression problem that we showcased for HEA property predictions. This technically distinguishes the AMA investigation as a distinct case study.

#### 3.2.1 Case Study Two: Glass-Forming Ability

The glass-forming ability of an alloy characterizes its potential to solidify into an amorphous state, such as a bulk metallic glass (BMG). Alternatively, it may solidify into an amorphous ribbon (referred to as a ribbon metallic glass, or RMG), or a crystalline alloy (CRA). The materials data set of glass-forming ability encompasses a total of 6471 unique alloy compositions, and includes 1211 BMGs, 1552 CRAs, and 3708 RMGs. In instances where multiple reports on the glass-forming ability of specific alloys arise, the highest reported glass-forming ability was consistently selected for inclusion in our analysis.

**Figure 8:**
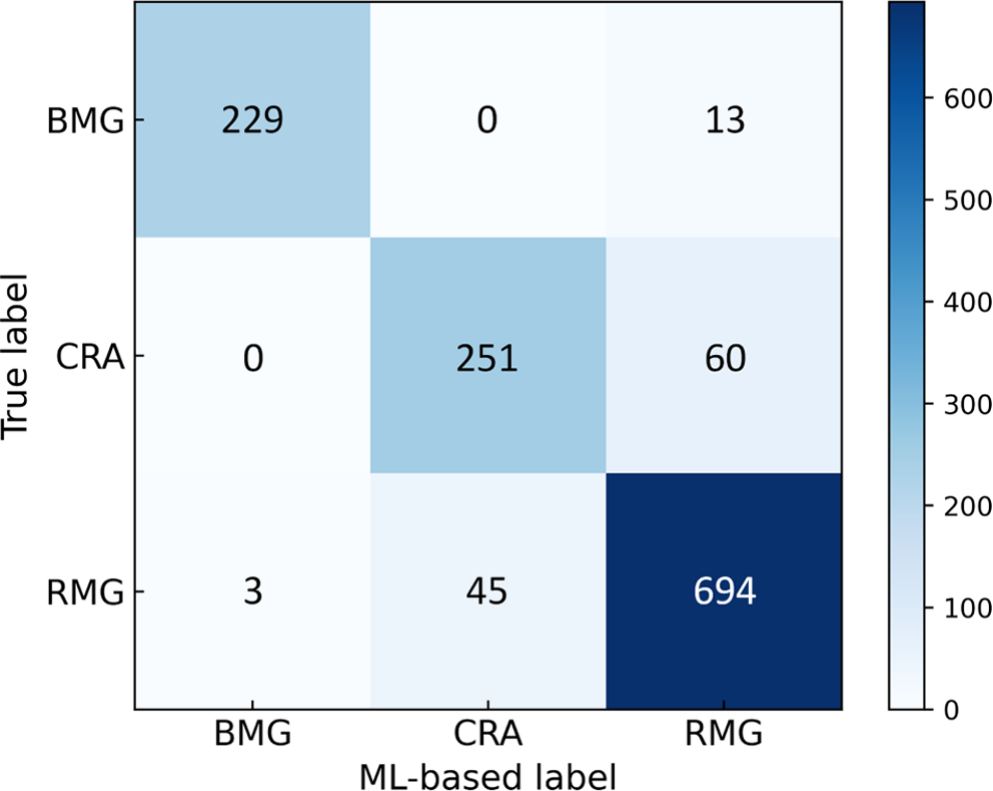
Confusion matrix for the classification of alloys by their glass-forming ability.

The classification model that we developed using the GBFS workflow demonstrated remarkable efficacy, outperforming several benchmarks in the literature. From an initial pool of ca. 650 exploratory features and ca. 130 engineered features, the model identified the 25 most pertinent features. It achieved an F1-score of 0.91, an AUC-ROC of 0.98, and an accuracy of 0.91. The extent to which the glass-forming abilities of these alloys have been correctly classified is delineated by the confusion matrix shown in Figure 8, with a summary of the corresponding precision, recall and F1-score evaluation metrics being provided in Table 4, while some of the most salient features identified by the GBFS workflow are given in Table 5.

**Table 4:** Summary of the Performance Metrics for the Classification of Alloys by Their Glass-Forming Ability

	precision	recall	F1-score
BMG	0.987	0.946	0.966
CRA	0.869	0.810	0.839
RMG	0.907	0.945	0.925
macro average	0.921	0.900	0.910
weighted average	0.913	0.913	0.912

**Table 5:** List of Most Relevant Features Identified by Our GBFS Workflow

no.	feature description
1	configuration entropy
2	mismatch of local radii
3	Miedema’s enthalpy of mixing
4	average deviation in Goldschmidt’s atomic volume per atom among the constituent elements of an alloy
5	range of the Mendeleev number among the constituent elements of an alloy
6	range of the coefficient of linear thermal expansion among the constituent elements of an alloy
7	average deviation of electronegativity among the constituent elements of an alloy
8	mean valence electron concentration (VEC)

Moreover, when applying a 10-fold cross-validation, our ML model realized an accuracy of 0.91 ± 0.01, a weighted AUC-ROC of 0.97 ± 0.01 and a weighted F1-score of 0.91 ± 0.01. In comparison, Xiong et al.^102^ reported an accuracy of 89.52% utilizing 36 features with a random-forest algorithm on the identical data set. This demonstrates the efficacy of our classification model developed via the GBFS workflow. Xiong et al. reported that the majority of their model performance is likely attributable to just 6 features. Our findings corroborate this, as we observed that the most significant loss reduction or variance gain was achieved with the first 5 features, particularly the top three, as depicted in Figure 10b. To explore this further, we retrained our predictive model using only the first 6 features listed in Table 5, which highlights the most relevant features that are identified by our GBFS workflow. This method resulted in a classification accuracy of 89%, indicating a clear consistency between the two studies. Therefore, it is evident that our modeling approach is on par with state-of-the-art methodologies.

Delving into specific metrics, precision quantifies the ratio of true positives relative to all positive predictions made by the model. For instance, a precision of 0.987 for BMGs implies that out of all the instances predicted as BMGs by the model, 98.7% were actually BMGs. Conversely, recall reflects the fraction of actual positive instances accurately identified by the model, with a recall of 0.946 for BMGs indicating that 94.6% of real BMGs were identified. The F1-score serves as an integral measure, amalgamating precision and recall into a singular metric via their harmonic mean. A high F1-score, approaching 1, denotes a perfect equilibrium between precision and recall. Notably, our model’s F1-score of 0.966 for BMGs denotes both high precision and recall, which is indicative of its robustness in being able to predict the glass-forming abilities of AMAs. For RMG classifications, the precision, recall, and F1-score surpass the 0.9 threshold, attesting to the highly discriminative nature of our classifier. While the performance metrics for CRA classification are adequate, they are the lowest among the metrics, hinting at a relatively higher incidence of both false positives and false negatives.

The corresponding macro and weighted averages reveal the discriminative prowess of our classification model. Thereby, the macro average, which computes average scores across all classes without regard to class size, indicates balanced, high-precision performance with a value of 0.921. In contrast, the weighted average takes into account the support of each class (i.e., the number of instances in each class). The corresponding weighted average precision of 0.913 means that, when considering the populations of the different classes, the model maintains high precision.

The proximity of the macro and weighted averages indicates that our model performs uniformly across the different classes. This high level of uniformity is likely to be attributable to our application of an oversampling technique in the training set such that there were equal representations of each class. Thereby, we employed random oversampling (ROS) of the training set to ensure equitable class representation by synthesizing feature vectors that resemble the characteristics of the minority classes within the high-dimensional feature space. Our implementation of ROS drew on a smoothed bootstrap resampling technique that effectively mitigated potential learning biases. This technique introduces a perturbation to the oversampled feature vectors by adding Gaussian noise that can be regulated by a smoothing matrix. This prevents the overfitting of a model on specific values of a feature, which helps to improve the generalization of the model.

#### 3.2.2 Gradient Boosted and Statistical Feature Selection Workflow

We now discuss the outcomes associated with each component of our GBFS workflow. While an exhaustive analysis was presented in the regression case study on HEAs, we succinctly outline the principal conclusions for AMAs and note any distinctions of relevance.

**Figure 9:**
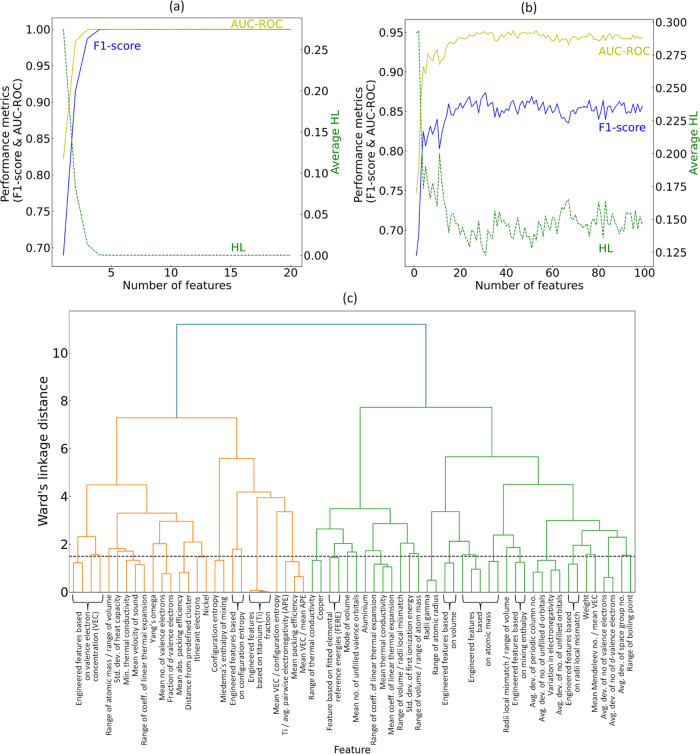
GBFS results for the prediction of glass-forming ability of AMAs. The figure shows the performance of GBDTs on (a) the training set and (b) the validation set, where classification models are trained recursively with an increasing subset of features, beginning from the most relevant feature based on the realized total loss reduction. (c) Multicollinearity reduction for the classification analysis, showing the dendrogram of the hierarchical agglomerative clustering using the remaining 64 features after performing the correlation analysis. The dashed horizontal line in black represents the distance threshold of 1.5 unit of Ward’s linkage distance.

As has been stated, our GBFS workflow short-listed a subset of 25 significant features from an original set comprising ca. 650 exploratory features and ca. 130 engineered features. The systematic refinement of the feature set during the recursive training of the GBDTs resulted in optimized model performance as evidenced by the convergence of key statistical metrics, including the F1-score, AUC-ROC, and Hamming loss (HL), with the feature-relevance ranking being determined by the aggregate loss reduction achieved during the training phase. This process is graphically represented in Figure 9, illustrating that performance metrics reach a plateau after incorporating ca. 5 of the most salient features in the training set, and ca. 35 in the validation set. As anticipated, the convergence trajectory for the validation set displayed greater fluctuation, consistent with the inherent variability of out-of-sample data. It should be reiterated that at this stage, interdependencies, such as multicollinearity among exploratory features, were not explicitly considered. Such multicollinearity could lead to an even distribution of the total loss reduction among correlated features, thereby concealing their true individual impact on the outcome variable.

A suite of feature analysis is ran in parallel to the recursive training of GBDTs, whose methodologies were distinct from those used in our regression study (cf. case study 1). Case study 2 employed a generalization of the one-way analysis of variance F-test, the Pearson’s chi-squared test, mutual information (MI) analysis, and discriminant analysis using logistic regression. Notable among the statistically significant features that were consequently identified are configuration entropy, the mean Mendeleev number of constituent elements, the minimum electronegativity, the range of atomic weights, and the range of Goldschmidt’s volume per atom among the constituent elements within the alloy. The selection process through these analyses, combined with those features effectuating maximal loss reduction in the prior recursive stage, informed the generation of new features. Employing a brute-force approach, we derived an additional 132 features, culminating in a comprehensive preliminary set of 182 features poised for the full classification analysis.

Advancing through the stages of our GBFS workflow, we tackled multicollinearity reduction, appraised the permutation importance of the refined features, and executed RFE to identify the ultimate feature subset for Bayesian optimization of our ML model.

**Figure 10:**
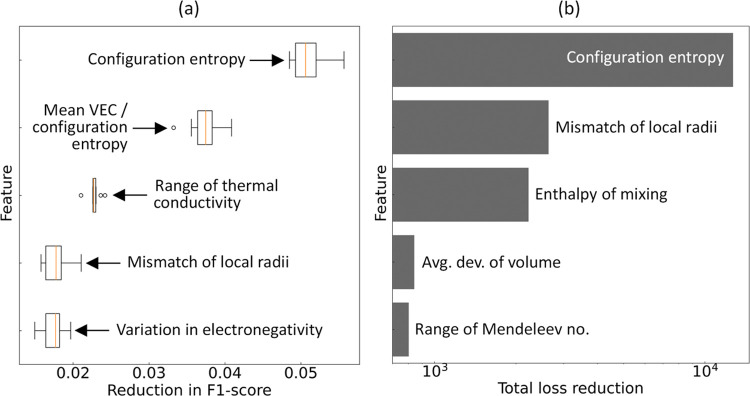
(a) Feature relevance and (b) permutation-based feature importance plots for the classification analysis of alloys by their glass-forming ability, displaying the five most significant features.

The two-step multicollinearity treatment systematically removed 143 features. Specifically, the correlation analysis eliminated 118 features whose correlation coefficient exceeded 0.8, while the hierarchical cluster analysis further excised 25 features, utilizing the Spearman rank-order correlation with a Ward’s linkage distance threshold of 1.5 units. This curation retained a subset of 39 features. The selection of the optimal distance threshold was informed by the Elbow method, and the resulting dendrogram, which visualizes the hierarchical agglomerative clustering, is displayed in Figure 9c. Furthermore, insights from the 10-fold permutation feature-importance evaluation, illustrated in Figure 10a, emphasize configuration entropy as the most relevant feature, which succeeds in significance the ratio of valence electron concentration to configuration entropy, the range of thermal conductivity, the mismatch of local radii, and variations in electronegativity among alloy constituents, presented in descending order of their permutation importance.

Consecutively, RFE was performed via a 10-fold cross-validation approach with weighted F1-score as the evaluation criterion; this distilled the feature pool down to a salient subset of 25 features. This final feature selection emerged from a comprehensive array of ca. 750 candidate features, signifying those of greatest relevance to the predictive objective, devoid of prior knowledge of the domain. Subsequently, to conclude our workflow sequence, we applied the optimization procedures described in our regression case study, to ascertain the architecture of our final predictive model for case study 2. Figure 10b shows the features that afforded the largest total loss reduction in predicting the target variable using the final classification model. The top 8 most significant features are listed in Table 5. These features largely concur with those discerned in preceding analyses of the GBFS workflow, underscoring the consistency of feature influence across different stages of model development.

#### 3.2.3 Feature Interpretation

Our GBFS workflow identified the most salient feature as the configuration entropy, which is a measure derived from the statistical distribution of different elements within an alloy. It quantifies the disorder associated with the arrangement of different types of atoms or ions in a material structure and is based on the probability of finding a particular element at a given atomic point. This is calculated by
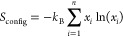
16where *k*_B_ is the Boltzmann constant, *x*_*i*_ is the mole fraction of the *i*-th component in the alloy, and *n* is the number of different elements in the alloy. Equation 16 quantifies the contribution of configurational disorder to the entropy of an alloy. This entropy is influenced by the diversity and distribution of constituent elements; specifically, it increases as the number of distinct elements rises and their distribution approaches equimolarity. In the context of BMGs, configurational entropy plays a pivotal role in facilitating the formation of amorphous structures. High configurational entropy, arising from a significant diversity of elements and their equitable distribution within the alloy, acts to suppress the crystallization process during cooling. This suppression helps to maintain the alloy in a glassy state by obstructing the orderly arrangement of atoms that typifies crystalline structures. Furthermore, elevated configurational entropy enhances the thermodynamic stability of these amorphous phases. This relationship is articulated through the Gibbs free energy equation:
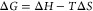
17where *G* represents the Gibbs free energy, *H* denotes the enthalpy, *T* is the temperature, and *S* signifies the entropy. An increase in entropy leads to a reduction of the Gibbs free energy for the amorphous phase, thereby favoring its formation over a crystalline phase. This principle underscores the preferential formation of the amorphous state in materials with high configurational entropy, which correlates with a greater glass-forming ability. The level of heterogeneity in atomic sizes and bonding characteristics among multiple elements serves to disrupt the crystal nucleation process and slow down grain growth, further favoring the development of amorphous structures over crystalline structures. Therefore, configuration entropy is pivotal in predicting the glass-forming ability of AMAs and ensuring their stability as noncrystalline solids.

The second salient feature was identified as the mismatch of local atomic radii, which relates to local variations in atomic sizes among the constituent elements of the alloy. In other words, it quantifies the extent to which the atomic radii of the elements that make up the material differ from each other, affecting the physical and chemical properties of the alloy. A significant mismatch in the atomic radii among the constituent elements disrupts the regular atomic packing required for the formation of crystalline phases. This disruption impedes the nucleation and growth of crystal lattices, thereby enhancing the propensity of the alloy to form an amorphous structure. In addition, the variability in local atomic radii among the constituent elements typically elevates the viscosity of the melt. This increased viscosity impedes the atomic diffusion and rearrangement necessary for crystal growth, thereby enhancing the glass-forming ability of the alloy. This phenomenon can be conceptualized within the framework of energy landscape theory, where variations in atomic sizes contribute to a complex energy landscape with more local minima. These minima can entrap the structure in a noncrystalline, amorphous state as it cools. Consequently, an understanding and ability to manipulate this feature can significantly impact the ability to produce materials with high glass-forming ability.

The third prominent feature contributing significantly to the reduction in loss within our model is the calculated enthalpy of mixing (Δ*H*) for an alloy, as determined by Miedema’s model.^103^ Miedema’s model, a foundational semiempirical methodology in materials science, forecasts the formation enthalpies of alloys. It incorporates variables such as disparities in atomic sizes, electronegativity, and electron densities of constituent elements to estimate the enthalpic changes when different metals amalgamate to form an alloy. This predictive capability is crucial for understanding and engineering the thermodynamic properties of new alloy compositions.

More specifically, Miedema’s model, through the Δ*H* parameter, predicts the thermodynamic nature of mixing among various metallic elements, discerning whether the interaction will be exothermic or endothermic. A negative value of Δ*H* (i.e., exothermic mixing) generally indicates that the components of the alloy are likely to form a stable solution or an amorphous phase, rather than segregating or crystallizing into intermetallic compounds. This inherent stability is conducive to the formation of amorphous structures. Consequently, alloys characterized by more negative values of enthalpy of mixing typically exhibit a higher glass-forming ability, as these favorable energy states can facilitate the bypassing of crystallization pathways during the cooling process. Alloys characterized by a significantly negative enthalpy of mixing typically exhibit increased viscosity in their molten state. This increased viscosity restricts atomic mobility which is essential for the formation of crystalline structures, thereby increasing the likelihood of glass formation as the alloy cools. Δ*H* appears to be a critical parameter for predicting and understanding the glass-forming ability of alloys. It provides a theoretical basis for anticipating how different elements will interact thermodynamically, influencing the stability and formability of amorphous phases. See Table 5 for additional features that are deemed pertinent in the classification of alloys by their glass-forming ability.

#### 3.2.4 Predicting Other Properties of AMAs

**Figure 11:**
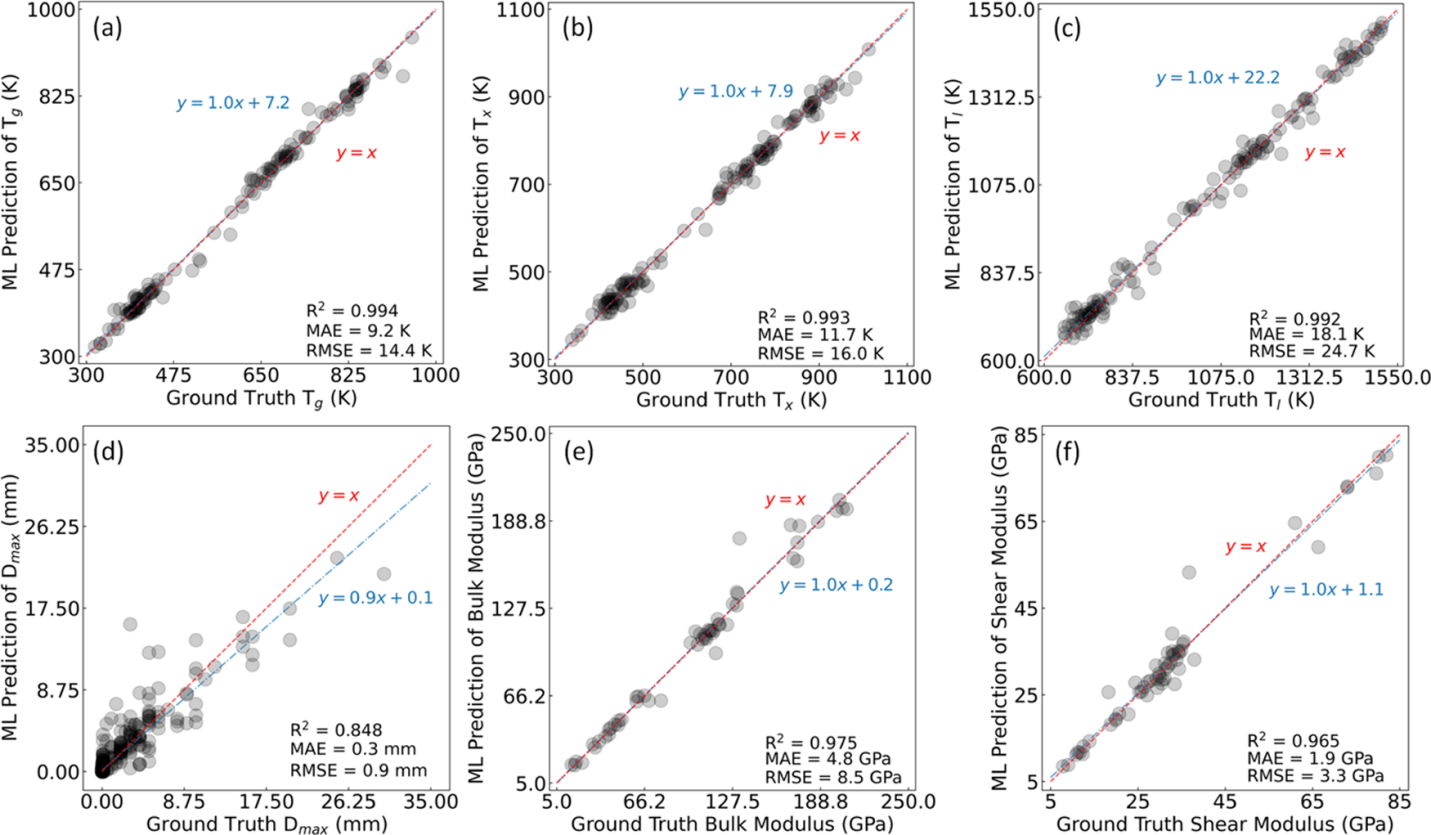
Property predictions of AMAs. The dashed red line is drawn to represent the hypothetical case, where the ML-based prediction would equal the literature values. The blue dot-dash lines are linear fits generated using OLS method, applied to various materials properties: (a) glass transition temperature *T*_g_, (b) onset of crystallization temperature *T*_x_, (c) liquidus temperature *T*_l_, (d) critical casting diameter *D*_max_, (e) bulk modulus, and (f) shear modulus.

Having established a GBFS-based model for predicting the glass-forming ability of AMAs, we broadened the application of our modeling approach to create and train individual models that are capable of predicting a wider range of properties of AMAs; this includes characteristic transformation temperatures (CTTs), the critical casting diameter (*D*_max_) and elastic moduli. The results are displayed in Figure 11, while the corresponding error distributions are presented in Supporting Information 5.

The results pertaining to the prediction of CTTs are presented in Figure 11a–c and Figure S5a–c, where *T*_g_ denotes the glass transition temperature, *T*_x_ the onset of crystallization temperature, and *T*_l_ the liquidus temperature. CTTs are crucial for establishing criteria to evaluate the glass-forming ability of alloys.^104^ The ground-truth values were derived from 674 measurements conducted via differential thermal analysis or differential scanning calorimetry at a consistent heating rate, primarily at 20 K/min. Different combinations of transformation temperatures can relate *D*_max_ to glass-forming ability criteria. Notably, this includes the supercooled liquid range (Δ*T* = *T*_x_ – *T*_g_)^104^ and the reduced glass transition temperature (*T*_rg_ = *T*_g_/*T*_l_).^105,106^ Hence, CTTs serve as critical parameters in the design and characterization of AMAs.

The predictive outcomes for *D*_max_ are depicted in Figure 11d and Figure S5d. *D*_max_ is the largest diameter or section thickness of an alloy that can be cast into a fully amorphous rod or plate. The ground-truth data set for *D*_max_ includes measurements for 5934 alloys, incorporating only the critical copper-mold casting diameter values reported in the literature for BMGs. Generally, a slower cooling rate allows for a larger *D*_max_, indicating a higher glass-forming ability, where the critical cooling rate is defined as the slowest cooling rate at which an alloy can solidify without crystallization. Consequently, *D*_max_ serves as another important parameter for assessing the glass-forming ability of alloys.

The predictive results for the shear and bulk moduli of BMGs are presented in Figure 11e,f and Figure S5e,f. The ground-truth data set comprises 278 unique BMGs, with their moduli measured using resonant ultrasonic spectroscopy. For cases where multiple measurements are reported in the literature, the mean value has been computed. These elastic moduli, or the ratios thereof, are critical mechanical properties that influence the glass-forming ability and the overall behavior of AMAs.^75^ The shear modulus plays a pivotal role in theoretical models that assess the glass-forming ability of alloys. For instance, models such as the fragility index of glasses utilize the temperature dependency of the shear modulus to predict how easily a glassy state forms under varying cooling conditions. A lower shear modulus at high temperatures can indicate a greater likelihood for glass formation. Conversely, a higher bulk modulus indicates a lower compressibility of the alloy, which typically correlates with increased density and potential stability of the glass. This enhanced stability is essential for preventing the crystallization of the amorphous structure under various thermal and mechanical stresses.

Having broadened our analysis to encompass multiple target properties, it is crucial to conduct a comparative evaluation of our predictive capabilities against those documented in the existing literature. We align our results with state-of-the-art models reported in scholarly articles, employing identical data sets for these properties to facilitate a direct comparison.

In our examination of the elastic properties of AMAs, we conducted a detailed comparison with the findings of Xiong et al.^102^ Their work, an important benchmark in the field, reported an RMSE of 3.6 GPa and an *R* value of 0.98 for shear modulus predictions, alongside an RMSE of 9.5 GPa and an *R* value of 0.98 for bulk modulus predictions. These metrics underscore their model’s high accuracy and the robustness of their predictive analytics. In comparison, our ML models demonstrated comparable performance. Thereby, for the shear modulus, our models achieved an RMSE of 3.3 GPa and maintained an *R* value of 0.98 (or an *R*^2^ of 0.97), indicating precise predictions with minimal variance. More notably, for bulk modulus predictions, our models showed an improved RMSE of 8.5 GPa and a *R* value of 0.99 (or an *R*^2^ of 0.98). This indicates a statistically significant fit to the data, underscoring the efficacy of our modeling approach in accurately capturing the underlying patterns in the materials data. We also observe that the linear fits produced using the OLS method demonstrate strong consistency between the predicted values and the ground truth, as both target properties yielded a gradient of 1.0. There is a positive bias of 1.1 and 0.2 GPa at lower property values for shear and bulk modulus, respectively. However, the impact on the predictions appears minimal, especially within the range of feature values considered in this analysis.

Shifting our attention to CTTs, which are pivotal in assessing the glass-forming ability of alloys, our models demonstrated remarkable accuracy. Notably, the highest *R*^2^ value achieved was 0.994 for predicting *T*_g_, whereas the prediction of *T*_l_ achieved a slightly lower, yet still impressive, *R*^2^ value of 0.992. These high values of *R*^2^ indicate excellent model performance in predicting key thermal transitions in alloy systems. Upon a closer analysis on the most influential exploratory features within these predictions, the mean melting temperature of the constituent elements in a given alloy consistently emerged as the most critical predictor of CTTs. This feature significantly contributed to loss reduction and variance gain, illustrating its central role in the models’ ability to accurately predict CTTs. Following closely was the mode of the ground-state volume per atom among the constituent elements, which also played an important role in the predictive process. Additionally, the standard deviation of the velocity of sound traveling through these elements proved to be another key feature, reflecting how acoustic properties correlate with various forms of thermal behavior in materials. These findings align closely with those reported by Xiong et al.,^102^ who also highlighted the predominant influence of the mean melting temperature on CTTs, noting an R value greater than 0.93 in correlation with CTT target values. Their findings similarly emphasized the importance of atomic radius, which align with our observations regarding the significance of the volume per atom. Such consistent results across different studies reinforce the reliability of these features as predictors of alloy behavior, particularly in the context of glass-forming ability, and underscore the interconnected nature of thermal, acoustic, and volumetric properties in materials science.

The final target property we will discuss in this analysis is *D*_max_. Our predictive model for this target property achieved an *R* of 0.92 (or an *R*^2^ of 0.85) and an RMSE of 0.9 mm. The linear regression, executed using the OLS method, presented a gradient of 0.9 and a positive bias of +0.1. The scatter plot of this regression reveals a relatively dispersed set of data points compared to other properties that we have examined, illustrating a variability in predictive accuracy across the data set. Notably, the majority of the *D*_max_ values were observed to be below approximately 9 mm. This distribution characteristic significantly contributes to the lower model accuracy for larger *D*_max_ values, where deviations become more pronounced. When comparing our results with those documented in the literature,^102^ where an RMSE of 1.2 mm and an *R* value of 0.85 were reported for the same target property, our predictions show close concordance. These results affirm the robustness of our ML models, which were refined through our GBFS workflow in a systematic manner, without requiring any human intervention in selecting the feature space. This automated approach proves highly effective in capturing and predicting diverse material properties, emphasizing the capability of ML to adapt to and accurately model complex patterns within materials data.

We further analyzed the chemical compositions that are associated with the extended tails in the error distributions for CTTs and *D*_max_ as detailed in Supporting Information 5. For *T*_g_, numerous alloys prominently incorporate rare earth elements such as La, Pr, Gd, and Nd. These elements are strategically utilized in alloys to improve properties such as corrosion resistance and high-temperature strength. Additionally, transition metals such as Ni, Cu, and Zr, along with refractory metals such as Nb and Ta, are frequently included in various compositions. Al also features prominently in several alloys, indicating an intention to exploit its lightweight characteristics and its capacity to form amorphous structures when alloyed with larger atoms from rare earth elements.

In the analysis of *T*_x_ and *T*_l_, we observe a similar chemical profile among the outliers, which is characterized by a significant use of rare earth elements (e.g., La, Ce, Pr, Ho, and Y). These elements are used for their distinctive properties, including enhanced stability at high temperatures and resistance to oxidation, which are typical benefits of rare earth metals. For instance, chemical elements such as La and Ce are incorporated in alloys to refine their grain structure, thereby enhancing the mechanical properties of alloys. The presence of multiple principal elements in diverse ratios suggests an approach that is aimed at exploiting the synergistic effects among different metals. The inclusion of Al and Mg in various alloys highlights its role in reducing overall weight while simultaneously improving mechanical strength.

When examining the chemical compositions associated with the heavy tail in the error distributions for *D*_max_, a consistent chemical trend emerges. This encompasses the use of rare earth and exotic metals such as Nd and Gd, along with Al and Mg to optimize strength-to-weight ratios. A notable feature is the prevalent use of Cu and Zr, which are important chemical elements in the formation of BMGs. Additionally, there is a frequent inclusion of Ag, which likely aims to enhance the durability of the alloys, while the inclusion of Be is likely due to its stiffness and thermal stability. Once more, the majority of these chemical compositions have been crafted with complex, multicomponent formulas that were designed to exploit the synergistic effects of various elements to enhance overall properties. Yet, this high level of complexity can introduce challenges in accurately predicting their properties.

We now examine the model predictions by focusing on specific regions of interest that are relevant to various engineering applications, similar to our approach for the properties associated with HEAs. In general, the calculated performance metrics (i.e., *R*^2^, MAE, and RMSE) provide a good indication of the model performance across their respective ranges, showing minimal or consistent deviations from the ground truth for the CTTs and elastic moduli. However, a different pattern emerges for the prediction of *D*_max_, as illustrated in Figure 11d. Here, we observe a low prediction error within regions below 7 mm. In contrast, the model accuracy diminishes for predictions concerning *D*_max_ values that are greater than 7 mm, particularly when *D*_max_ exceeds 10 mm. This observation suggests that for applications that require materials with large *D*_max_, the effectiveness of the model should be assessed based on its accuracy within this higher range. Therefore, it is imperative to evaluate the predictions by focusing on specific regions of interest for applications, beyond just the overall model accuracy.

For AMAs (or BMGs), the desired *D*_max_ value is ideally as large as possible. This critical measurement reflects the maximum thickness at which the alloy can be cast and still maintain its amorphous structure without undergoing crystallization as it cools. This indicates that the model predictions may not be reliable for designing materials with a target *D*_max_ value that exceeds approximately 7 mm. It is important to make this limitation clear to the reader to ensure transparency regarding the capabilities of the model developed through our proposed GBFS workflow. The reduced performance at higher values can be attributed directly to the scarcity of data points available within this range. When directly comparing our results with those of Xiong et al.,^102^ who employed the same data set and established the benchmark for this study, we noted similar trends at higher values. Xiong et al. implemented various methodologies, from linear regression to random forest, achieving their best prediction of *D*_max_ with an *R* of 0.85 and an RMSE of 1.2 mm. In contrast, our model achieved an *R* of 0.92 (or *R*^2^ of 0.85) and an RMSE of 0.9 mm. Specifically, in predicting *D*_max_ values that are greater than 7 mm, our model exhibits comparatively smaller deviations. This observation suggests that there are added advantages to adopting a systematic approach to feature analysis, selection, modeling, and optimization, as proposed in this study. With the availability of more data in this range, we anticipate significant improvements in both modeling approaches.

## 4 Conclusions

This study has implemented a machine-learning-based workflow for feature selection and statistical analysis to train predictive models within the domain of high-entropy alloys (HEAs) and amorphous metallic alloys (AMAs). Our workflow has harnessed a distributed gradient boosting framework complemented by exploratory data and statistical analyses, as well as multicollinearity treatments. This methodology has efficiently identified and selected a subset of features that are highly relevant to the target variable or class within a multifaceted feature space, ensuring both minimal redundancy and maximum relevance. Gradient boosting trees were trained with the selected features, that were are derived solely from the chemical composition of a material. The robustness and effectiveness of our modeling approach has been illustrated through two case studies on two types of alloys: (i) a regression analysis of the bulk modulus of HEAs, and (ii) a classification analysis of AMAs based on their glass-forming ability. In the prediction of bulk modulus for HEAs, our Bayesian-optimized regression model demonstrated exemplary performance on the test set, achieving an *R*^2^ of 0.969, an MAE of 3.958 GPa, and an RMSE of 5.411 GPa. Similarly, for predicting the glass-forming ability of AMAs, our Bayesian-optimized classification model reached an F1-score of 0.91, an AUC-ROC of 0.98, and an accuracy of 0.91. These outcomes are generally superior to those reported in existing literature, including some studies that employ more intricate feature descriptors. By leveraging chemical data compiled from a diverse range of literature sources, we have successfully predicted a wide range of properties for alloys. This demonstration not only confirms the efficacy of our modeling approach but also highlights the significance of comprehensive feature analysis and judicious feature selection, over a mere reliance on complex modeling.

## Data Availability

We have made available the code for the feature selection, statistical analyses, multicollinearity reduction, recursive feature elimination and Bayesian optimization at https://github.com/Songyosk/AlloysML. The data sets used in this work are made available as a part of the SI.

## Supporting Information Available

The uncertainty quantification associated with predictions of the HEA bulk modulus, the distribution of absolute errors for the property prediction of HEAs, the confusion matrix for the classification of HEAs by their experimentally observed crystal structures, and the distribution of absolute errors for the property predictions of AMAs (PDF)
The collated data and the corresponding chemical compositions are provided in AlloysDB.xlsx (XLSX)
