# Supplementary material for: Predictive Modeling of High-Entropy Alloys and Amorphous Metallic Alloys Using Machine Learning
Source: J Chem Inf Model. 2024 Oct 1;64(19):7313–36. doi: 10.1021/acs.jcim.4c00873 (PMC11480990; doi:10.1021/acs.jcim.4c00873)
Supplement: Supplementary file 1 — ci4c00873_si_001.pdf [file ci4c00873_si_001.pdf]

Supporting Information

Predictive Modeling of High-Entropy Alloys and Amorphous Metallic Alloys Using Machine Learning

Son Gyo Jung[1,2,3], Guwon Jung[1,3,4], Jacqueline M. Cole[1,2,3,*]

[1] *Cavendish Laboratory, Department of Physics, University of Cambridge, J. J. Thomson Avenue, Cambridge, CB3 0HE, UK*

[2] *ISIS Neutron and Muon Source, STFC Rutherford Appleton Laboratory, Harwell Science and Innovation Campus, Didcot, Oxfordshire, OX11 0QX, UK*

[3] *Research Complex at Harwell, Rutherford Appleton Laboratory, Harwell Science and Innovation Campus, Didcot, Oxfordshire, OX11 0FA, UK*

[4] *Scientific Computing Department, STFC Rutherford Appleton Laboratory, Harwell Science and Innovation Campus, Didcot, Oxfordshire, OX11 0QX, UK*

[*] jmc61@cam.ac.uk

SI. 1 Prediction of HEA bulk modulus: Uncertainty Quantification

Having employing cross-validation techniques to evaluate model uncertainty for the prediction of HEA bulk modulus, we now conduct a more detailed uncertainty quantification. Specifically, we apply an uncertainty estimation technique for regression and employ the calibration method outlined by Palmer et al.,[1] implemented through the Materials Simulation Toolkit for Machine Learning (MAST-ML).[2] However, our GBFS framework is not fully compatible with MAST-ML. Therefore, we have carried out the analysis by substituting our final machine-learning model with a random-forest algorithm from Scikit-learn,[3] which allows us to proceed despite the compatibility issues.

In this methodology, we began our analysis using an ensemble model, wherein individual predictions were computed from each sub-model that comprises the ensemble. This process led to the calculation of both the mean and the standard deviation of these predictions. The standard deviation was then calibrated to produce refined uncertainty estimates. To facilitate this, repeated five-fold cross-validation was systematically employed to generate the necessary residuals and standard deviation values for the calibration process. The outcomes of this process were then analyzed to assess the quality of the error bars, both pre- and post-calibration.

Figure S1.1 displays the distribution of z-scores for all left-out test data. Before calibration, the mean and standard deviation of the z-scores for equimolar quaternary HEAs are 0.015 and 0.703, respectively, while for non-equimolar quaternary HEAs, these values are 0.014 and 0.825, respectively. Post-calibration, the mean and standard deviation of the z-scores refined to 0.023 and 0.998 for equimolar quaternary HEAs, and to 0.019 and 0.996 for non-equimolar quaternary HEAs, respectively. The z-score distribution is expected to resemble a standard normal distribution, which is defined by a mean of zero and a standard deviation of one. For both types of quaternary HEAs, the recalibration of the error bars has effectively nudged the alignment of the z-score distributions closer to this standard normal ideal, particularly in the observed variance. Nevertheless, it is worth highlighting that a heavy-tailed distribution is present for the non-equimolar quaternary HEAs. This observation aligns with expectations outlined in our manuscript, where we incorporated an additional 132 non-equimolar quaternary HEA compositions into the cross-validation process. These compositions are distinct and not previously

included in the original training set. Such a finding underscores the significant influence of the level of dataset diversity on both model accuracy and error metrics.

Figure S1.2 presents an analysis of the quality of correlation between the true and predicted errors and examines the impact of calibrating error bars to enhance this correlation (i.e., to improve the accuracy of error predictions). Figure S1.2 shows a plot comparing the reduced root-mean-square residuals with the binned reduced-model-error estimates. This plot serves as a direct evaluation of how closely the true error aligns with the predicted error, on average. In an ideal scenario, the predicted error would align perfectly with the true error, as characterized by a slope and intercept of one and zero, respectively. Before calibration, for equimolar quaternary HEAs (Figure S1.2 (a)), the slope and intercept are 0.77 and -0.03, respectively, suggesting that the uncalibrated error bars tend to overestimate the true error. After calibration, the slope and intercept adjust to 1.08 and -0.02, respectively, marking a significant improvement in the correlation between true and predicted errors. This enhancement suggests that calibration effectively improves the distribution of z-scores, leading to a more accurate error prediction.

In Figure S1.2 (b), which focuses on non-equimolar quaternary HEAs, the slope and intercept before calibration are noted to be 0.79 and 0.02, respectively. Following calibration, the slope sees a small improvement, rising to 0.83. This is evident from the linear fit being closer to the diagonal line. Conversely, the intercept increases to 0.06, and the R^2 value drops from 0.96 to 0.93. This reduction in R^2 may suggest a sightly diminished ability to predict the variance in the dependent variable from the independent variable. Despite these subtle variations, the error bars for non-equimolar quaternary HEAs consistently overestimate the actual true error, both before and after calibration. This indicates that accurately predicting the bulk modulus and quantifying error margins for non-equimolar quaternary HEAs are comparatively more difficult.

Overall, this analysis concentrates on assessing the distribution of z-score values and the effectiveness of calibrating error bars to achieve a more favorable z-score distribution; that is, a error distribution that is closer to a standard normal distribution.

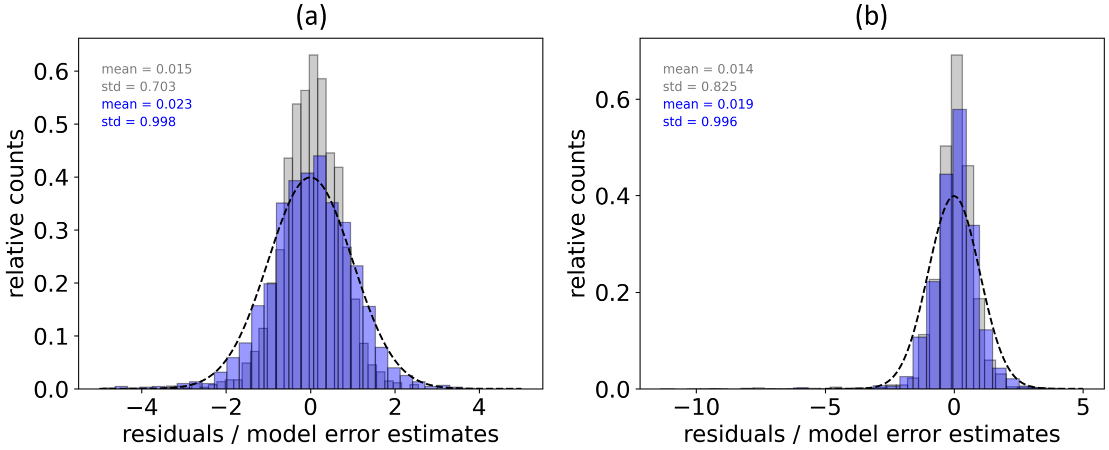

Figure S1.1: Distribution of z-scores over all left-out test data for (a) equimolar quaternary HEAs and (b) non-equimolar quaternary HEAs, before calibration (grey) and after calibration (blue).

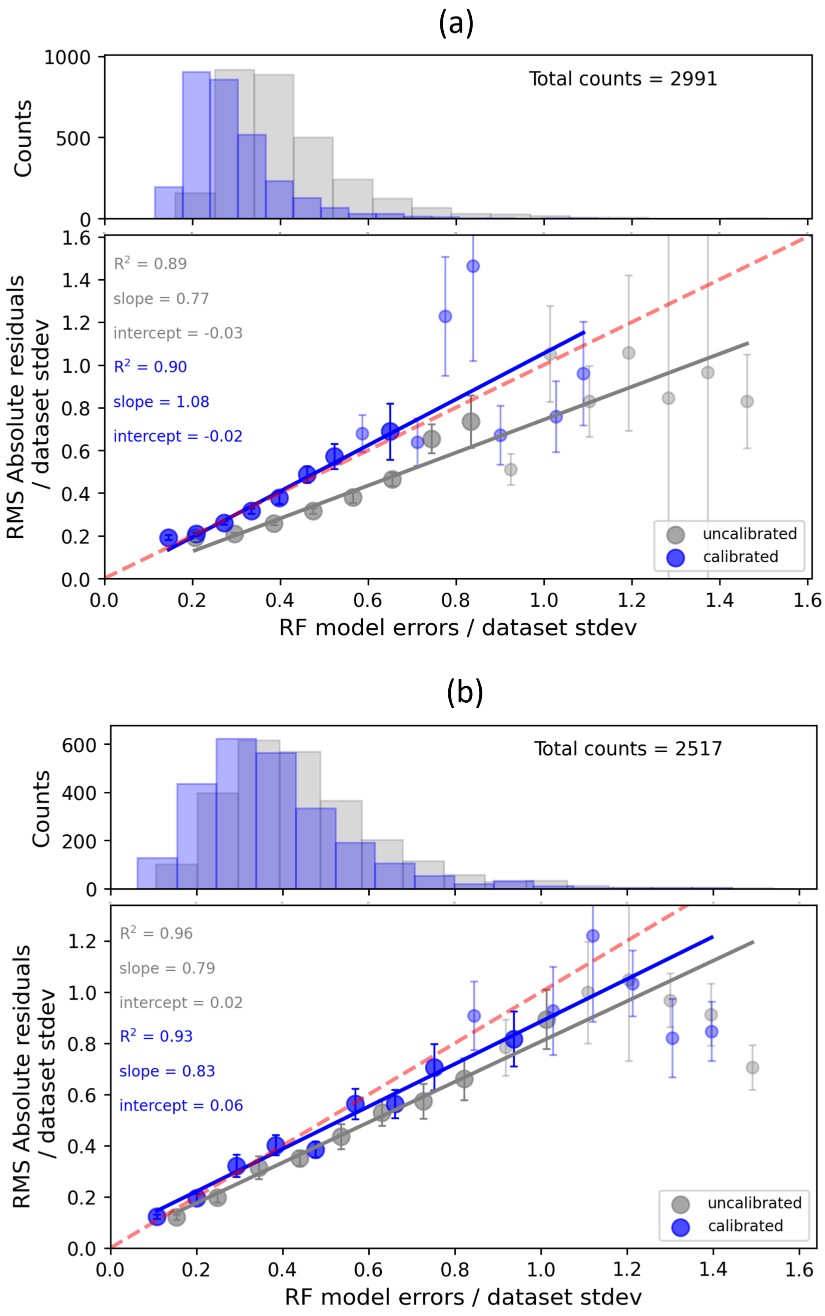

Figure S1.2: Plot of the reduced root-mean-square residuals (RMS) against binned reduced-model-error estimates for (a) equimolar quaternary HEAs and (b) non-equimolar quaternary HEAs, before calibration (grey) and after calibration (blue). The histograms illustrate the distribution of data points within each bin. The smaller and more transparent points correspond to the bins that lack sufficient data for reliable sampling, and therefore, are unlikely to provide accurate RMS residuals. The dashed diagonal line in red illustrates the scenario where the RMS residuals are equal to the binned-reduced-model error estimates.

SI. 2 Distribution of Absolute Errors for the Property Prediction of HEAs

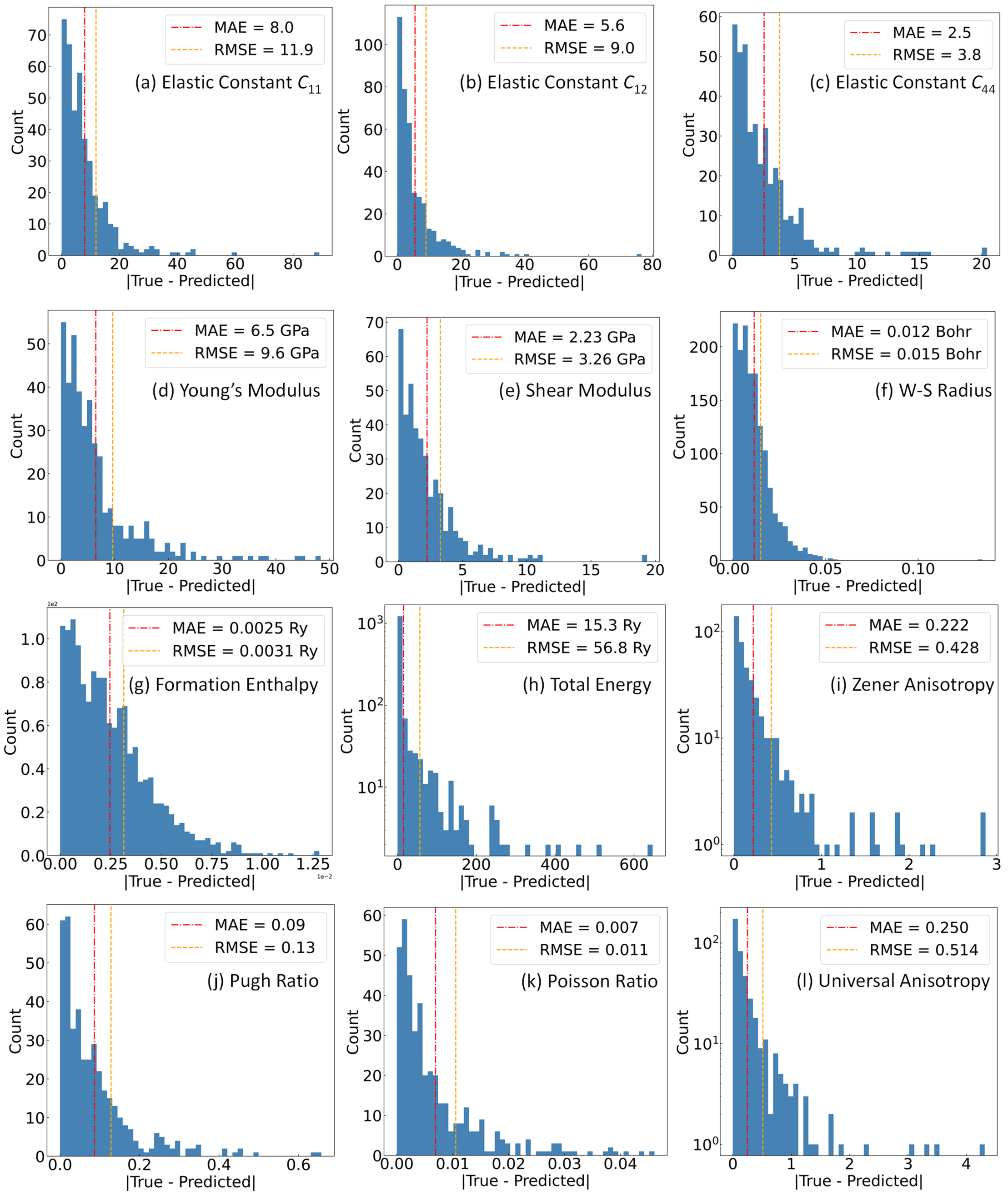

Figure S2: Distribution of absolute errors for the property prediction of HEAs, where the dashed line (−.) in red indicates the MAE and the dashed line (−−) in orange indicates the RMSE.

SI. 3 Confusion Matrix for the Classification of HEAs by Their Experimentally Observed Crystal Structures

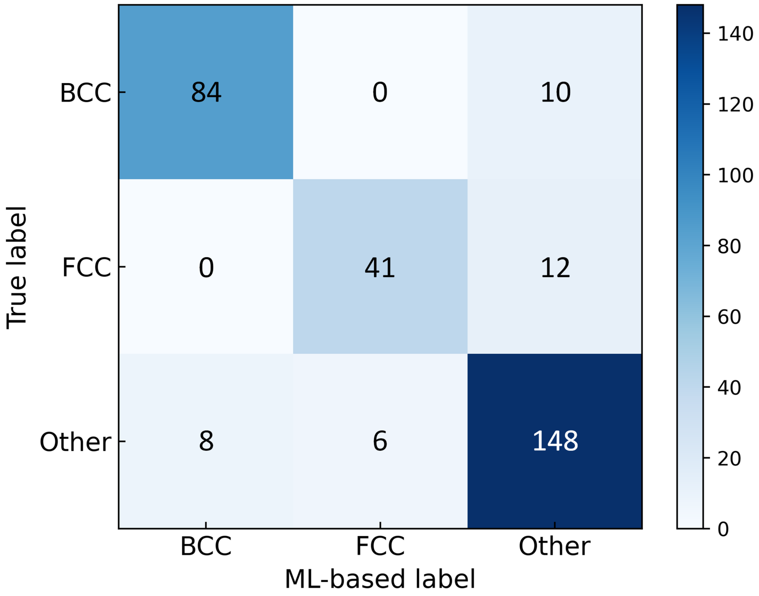

Figure S3: Confusion matrix for the classification of HEAs by their experimentally observed crystal structures,[4] which are classified into face-centered cubic (FCC), body-centered cubic (BCC) or other structural symmetries.

SI. 4 Boxplots of Model Accuracy for Four HEA Properties

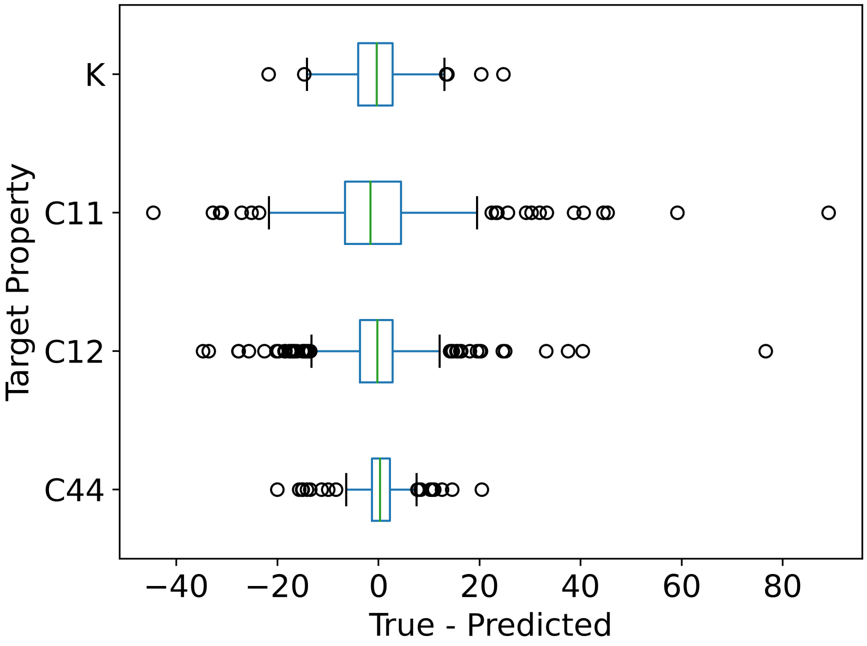

Figure S4: Boxplots illustrating the deviation of predictions from the ground truth for four HEA properties, namely bulk modulus (K in GPa) and elastic constants (C_{11}, C_{12} and C_{44}).

SI. 5 Distribution of Absolute Errors for the Property Predictions of AMAs

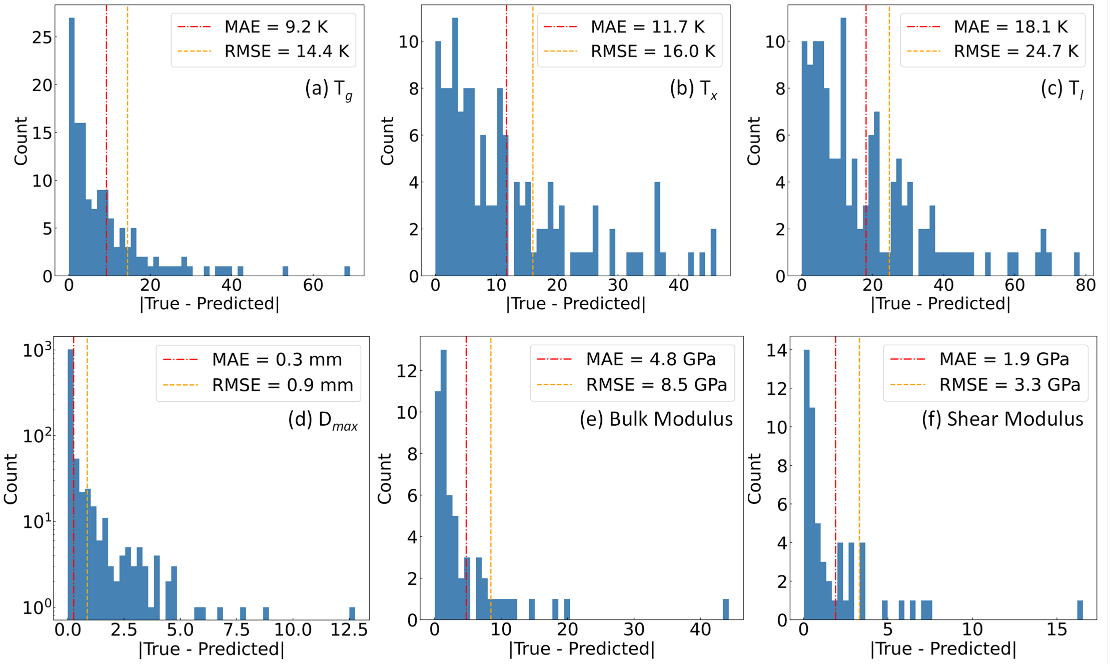

Figure S5: Distribution of absolute errors for the property predictions of AMAs, where the dashed line (−.) in red indicates the MAE and the dashed line (−−) in orange indicates the RMSE.

References

[1] Palmer, G.; Du, S.; Politowicz, A.; Emory, J. P.; Yang, X.; Gautam, A.; Gupta, G.; Li, Z.; Jacobs, R.; Morgan, D. Calibration after bootstrap for accurate uncertainty quantification in regression models. *Npj Comput. Mater.* **2022**, *8*, 115.

[2] Jacobs, R.; Mayeshiba, T.; Afflerbach, B.; Miles, L.; Williams, M.; Turner, M.; Finkel, R.; Morgan, D. The Materials Simulation Toolkit for Machine learning (MAST-ML): An automated open source toolkit to accelerate data-driven materials research. *Comput. Mater. Sci.* **2020**, *176*, 109544.

[3] Kramer, O.; Kramer, O. Scikit-learn. *Mach. Learn. Evol. Strat.* **2016**, 45–53.

[4] Borg, C. K.; Frey, C.; Moh, J.; Pollock, T. M.; Gorsse, S.; Miracle, D. B.; Senkov, O. N.; Meredig, B.; Saal, J. E. Expanded dataset of mechanical properties and observed phases of multi-principal element alloys. *Sci. Data* **2020**, *7*, 430.
